# Clinicopathologic Features of Mitochondrial Nephropathy

**DOI:** 10.1016/j.ekir.2021.12.028

**Published:** 2022-01-11

**Authors:** Toshiyuki Imasawa, Daishi Hirano, Kandai Nozu, Hiroshi Kitamura, Motoshi Hattori, Hitoshi Sugiyama, Hiroshi Sato, Kei Murayama, J-SMiN Collaborators

**Affiliations:** 1 Department of Nephrology, National Hospital Organization Chiba-Higashi National Hospital, Chiba, Japan; 2 Department of Pediatrics, The Jikei University School of Medicine, Tokyo, Japan; 3 Department of Pediatrics, Kobe University Graduate School of Medicine, Kobe, Japan; 4 Department of Clinical Pathology, National Hospital Organization Chiba-Higashi National Hospital, Chiba, Japan; 5 Department of Pediatric Nephrology, Tokyo Women's Medical University, Tokyo, Japan; 6 Department of Human Resource Development of Dialysis Therapy for Kidney Disease, Okayama University Graduate School of Medicine, Dentistry and Pharmaceutical Sciences, Okayama, Japan; 7 Department of Internal Medicine, JR Sendai Hospital, Miyagi, Japan; 8 Center for Medical Genetics, Department of Metabolism, Chiba Children’s Hospital, Chiba, Japan

**Keywords:** clinicopathologic feature, mitochondria, mitochondrial nephropathy, m.3243A>G, national survey, prognosis

## Abstract

**Introduction:**

The clinicopathologic characteristics of nephropathy associated with mitochondrial disease (MD) remain unknown. We retrospectively analyzed a cohort of patients with proteinuria, decreased glomerular filtration rate, or Fanconi syndrome who had a genetic mutation confirmed as the cause of MD, defined as mitochondrial nephropathy.

**Methods:**

This nationwide survey included 757 nephrology sections throughout Japan, and consequently, data on 81 cases of mitochondrial nephropathy were collected.

**Results:**

The most common renal manifestation observed during the disease course was proteinuria. Hearing loss was the most common comorbidity; a renal-limited phenotype was observed only in mitochondrial DNA (mtDNA) point mutation and COQ8B mutation cases. We found a median time delay of 6.0 years from onset of renal manifestations to diagnosis. Focal segmental glomerular sclerosis (FSGS) was the most common pathologic diagnosis. We then focused on 63 cases with the m.3243A>G mutation. The rate of cases with diabetes was significantly higher among adult-onset cases than among childhood-onset cases. Pathologic diagnoses were more variable in adult-onset cases, including diabetic nephropathy, nephrosclerosis, tubulointerstitial nephropathy, and minor glomerular abnormalities. During the median observation period of 11.0 years from the first onset of renal manifestations in patients with m.3243A>G, renal replacement therapy (RRT) was initiated in 50.8% of patients. Death occurred in 25.4% of the patients during the median observation period of 12.0 years. The median estimated glomerular filtration rate (eGFR) decline was 5.4 ml/min per 1.73 m^2^/yr in the cases, especially 8.3 ml/min per 1.73 m^2^/yr in FSGS cases, with m.3243A>G.

**Conclusion:**

Here, we described the clinicopathologic features and prognosis of mitochondrial nephropathy using large-scale data.

## Graphical abstract

**Figure:**
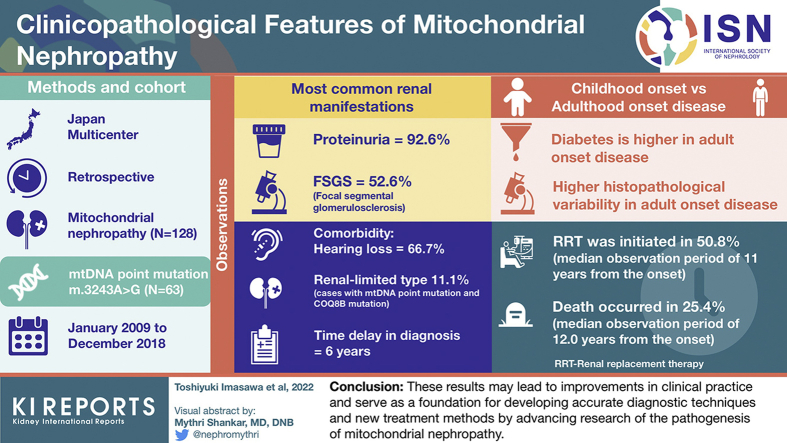

MDs are defined as diseases caused by a genetic defect in oxidative phosphorylation (OXPHOS) owing to mutations in genes in the nuclear DNA (nDNA) or mtDNA.^1^^,^^2^ The primary function of the mitochondria is to produce adenosine triphosphate through OXPHOS by the electron transfer system of the mitochondrial respiratory chain complex. As most of the energy required for cellular activities is supplied by the mitochondria in the form of adenosine triphosphate, MDs cause a variety of clinical manifestations in different organs.^3^ In addition, MDs are characterized by considerable phenotypic variability.^2^ MD can occur in both children and adults, and its symptoms are observed in various organs; there is even phenotypic variability in patients with the same gene mutation.^2^^,^^4^ For example, phenotypes and severity of MDs with mtDNA mutations such as m.3243 A>G vary depending on the heteroplasmy rates in the affected organs.^5^

Mitochondrial encephalopathy,^6^ myopathy,^7^ cardiomyopathy,^8^^,^^9^ hepatopathy,^10^ and deafness^11^ are caused by a genetic defect in the OXPHOS function in the cells of each organ. As reported previously, renal diseases with MD include FSGS, tubulointerstitial nephropathy, and Fanconi syndrome caused by proximal tubular dysfunction.^12^, ^13^, ^14^, ^15^, ^16^, ^17^, ^18^ Mitochondrial nephropathies can be caused by a genetic defect in OXPHOS function in cells composing each segment of the nephron, although it is difficult to biochemically evaluate decreased OXPHOS enzyme activities in actual cases. Thus, we propose that mitochondrial nephropathy is broadly defined as nephropathy with at least one of the following renal manifestations: proteinuria (≥0.15 g/g Cre), reduced eGFR (<60 ml/min per 1.73 m^2^), or Fanconi syndrome, along with a genetic mutation confirmed as the causative gene of MD.

A considerable number of case reports, case series, and review articles evaluating a collection of cases^12^, ^13^, ^14^, ^15^, ^16^, ^17^, ^18^, ^19^, ^20^, ^21^, ^22^ of mitochondrial nephropathy have revealed variability in mitochondrial nephropathy. Nevertheless, because of the rarity of this disease, the sample sizes evaluated in previous studies have been small, making it difficult to obtain an overall picture of mitochondrial nephropathy. A clinical study using large-scale data is necessary to understand and develop more accurate testing or treatment methods for mitochondrial nephropathy. We preliminarily analyzed the data of 38,351 cases registered in the Japan Renal Biopsy Registry^23^ from July 2007 to January 2018. Consequently, 16 nephropathy cases with MDs were extracted from the Japan Renal Biopsy Registry data. The number of cases was too small for a comprehensive analysis of mitochondrial nephropathy. Therefore, we recognized that a larger survey should be conducted, involving more nephrology sections throughout Japan, covering nephrology sections outside the Japan Renal Biopsy Registry, and including not only patients who underwent kidney biopsy but also those who did not. Here, we conducted a retrospective cohort study using a nationwide national survey in Japan, named J-SMiN.

## Methods

### Survey Process

As the first step to establish a cohort for the J-SMiN study, questionnaires pertaining to the total number of cases of nephropathy associated with MDs were sent to 757 nephrology sections registered with the Japan Renal Biopsy Registry (143 nephrology sections in Supplementary Summary S2) or certified as educational facilities by the Japanese Society of Nephrology (552 nephrology sections) or those in which the delegates of the Japanese Society for Pediatric Nephrology worked (62 pediatric nephrology sections). The inclusion criterion was all patients with nephropathy (decreased eGFR, proteinuria, and Fanconi syndrome) associated with MDs who visited a nephrology section from January 1, 2009, to December 31, 2018. The answered questionnaires were returned by 325 (42.9% of 757) nephrology sections in 46 of the 47 prefectures in Japan (Supplementary Summary S1). In 91 nephrology sections among the 325 sections, there were 159 cases of nephropathy associated with MDs. As the final step in establishing a cohort of the J-SMiN study, against these 91 nephrology sections, we conducted surveys to collect detailed data of each patient, with the permission of the ethics committee of each institute. The data collected included demographic, clinical, pathologic, and prognostic data.

### Definition of Mitochondrial Nephropathy

From the perspective of etiology, mitochondrial nephropathy should be defined as nephropathy caused by genetically defective OXPHOS function in cells comprising segments of nephrons. In this study, we broadly defined mitochondrial nephropathy as nephropathy with at least 1 of the following 3 renal manifestations: proteinuria (≥0.15 g/g Cre), reduced eGFR (<60 ml/min per 1.73 m^2^), and Fanconi syndrome, and with a genetic mutation already confirmed as the cause of MD. Although nephropathy should occur by nongenetic mitochondrial dysfunction as reported by us and others,^18^^,^^24^, ^25^, ^26^, ^27^, ^28^ here, we propose that such nephropathy with an acquired mitochondrial dysfunction should not be considered mitochondrial nephropathy, following the definition of MD, mitochondrial encephalopathy, mitochondrial cardiomyopathy, or mitochondrial myopathy.^1^^,^^2^^,^^6^, ^7^, ^8^, ^9^

### Estimated Glomerular Filtration Rate Calculations

If a patient's age at the time of measurement was ≥18 years, the eGFR was calculated using the serum creatinine level obtained using the Japanese equation (eGFR [ml/min per 1.73 m^2^] = 194 × age^−0.287^ × Cre^−1.094^ [× 0.739 if female]).^29^ If a patient was <18 years old at the time of measurement, the eGFR was calculated using the serum creatinine level obtained using the updated Schwartz equation (eGFR [ml/min per 1.73 m^2^] = 0.413 [height/Cre]).^30^ If a patient was <18 years old and lacked height data, the eGFR was calculated using the equation established by Hoste *et al.*^31^

### Determination of Onset Timing of Renal Manifestations

The J-SMiN questionnaire included questions on the presence of proteinuria (≥0.15 g/g Cre), reduced eGFR (<60 ml/min per 1.73 m^2^), Fanconi syndrome, and the age at which each of the manifestations was first detected. We considered the timing of the first detection of at least 1 of these 3 renal manifestations as the onset timing of renal manifestations. In addition, we evaluated cases in which age at the onset of renal manifestations was under 20 years as childhood-onset cases and cases in which the onset age was 20 years and above as adult-onset cases.

### Statistical Analyses

Continuous variables are expressed as median and interquartile range, whereas categorical variables are expressed as frequency. Univariate analyses were performed using Wilcoxon ranked sum test for continuous variables and the χ^2^ test or Fisher’s exact test for categorical variables, as appropriate. In addition, Kaplan–Meier curves were used to illustrate the time-to-event outcomes during the observation period after the onset of renal manifestations using the log-rank test to compare the results. Statistical analyses were performed using STATA software version 16.0 (StataCorp, College Station, TX), with *P* < 0.05 indicating statistical significance. The preplanned analysis was to compare the following 2 groups: childhood-onset and adult-onset groups of renal manifestations.

### Ethics

This study was approved by the independent ethics committee and institutional review board of Chiba-Higashi National Hospital (no. H31-19), the Japanese Society of Nephrology (number 65), and each center that participated in the National Survey of Mitochondrial Nephropathy in Japan (J-SMiN). This study was conducted in accordance with the principles of the Declaration of Helsinki.

## Results

### Description of the J-SMiN Cohort

**Figure 1:**
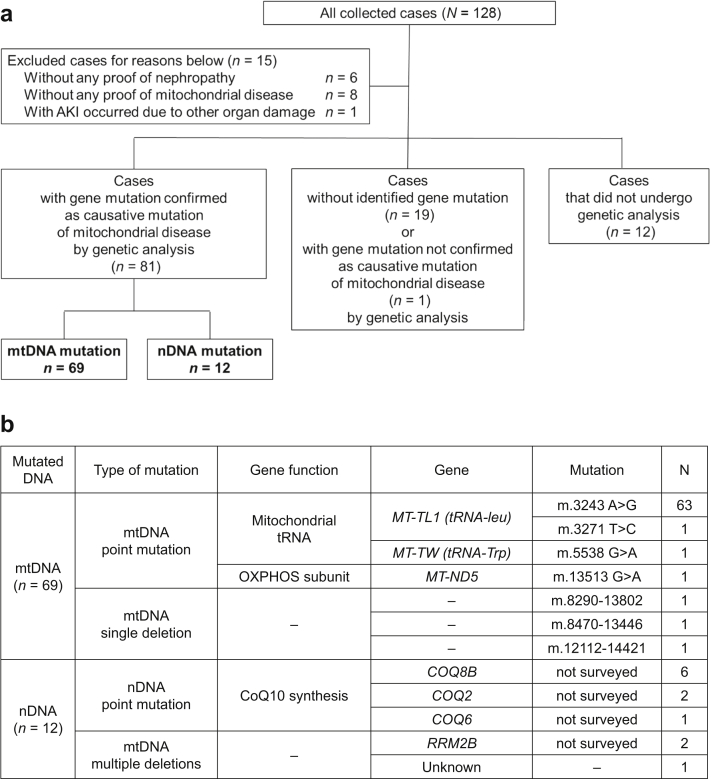
Diagram revealing information on the study participants and the summary of identified genes. (a) Diagram revealing the experimental results of the study participants. Cases without evidence of MD or nephropathy were excluded from the study. One case in which a worsening general metabolic status caused acute kidney injury was also excluded. Through genetic analysis, 81 cases were found to have gene mutation identified as the causative mutation of MDs. There were 20 cases without identified gene mutation that included 1 case with mtDNA mutation which has not been confirmed as the causative mutation of MDs. (b) Summary of identified causative gene mutations of MDs in this study. AKI, acute kidney injury; CoQ10, coenzyme Q10; MD, mitochondrial disease; mtDNA, mitochondrial DNA; nDNA, nuclear DNA; OXPHOS, oxidative phosphorylation; tRNA, transfer RNA.

Data of 128 cases were collected from 70 nephrology sections that participated in the J-SMiN study (Supplementary Summary S1). A schematic illustration of the reported cases is presented in Figure 1a. Finally, 81 cases with mtDNA mutations (*n =* 69) and nDNA mutations (*n =* 12) were analyzed following the definition of mitochondrial nephropathy in this study, as described in the Methods section. Figure 1b also reveals the identified mutated genes in cases included in this survey, including mtDNA point mutations, single or multiple deletions of mtDNA, and nDNA mutations related to coenzyme Q10 synthesis, along with the number of cases.

### Clinical Features of Mitochondrial Nephropathy

The clinical characteristics of each type of gene mutation are summarized in Table 1. A family history of MD was found in approximately half of the patients. Although patients with coenzyme Q10-related nDNA mutations had a family history in approximately half of the cases despite autosomal recessive inheritance, it involved the patients’ siblings. The most common renal manifestation observed during the course of the disease was proteinuria in 92.6% of the cases. For complications in organs other than the kidney, hearing loss (66.7%) was the most common complication, followed by diabetes (48.2%) among all cases, whereas developmental disorders were frequently observed in patients with *COQ2* mutation, *COQ6* mutation, and mtDNA single deletion. Retinitis pigmentosa, optic atrophy, pancreatitis, arrhythmia, liver dysfunction, growth hormone deficiency, hypothyroidism, hypoparathyroidism, depression, and autism, all of which have been reported to occur in MDs, were reported, with frequencies of <10% (Supplementary Table S1). The “renal-limited” type, in which there were no comorbidities in organs other than the kidneys, was observed in 11.1% of the cases and such a renal-limited phenotype was observed only in mtDNA point mutation and COQ8B mutation cases (Table 1). The median age of onset of renal manifestations, either decreased eGFR, proteinuria, or Fanconi syndrome, across all mitochondrial nephropathies collected in this study, was 19.0 years. The study also revealed that the current median time from the onset of renal manifestations to genetic diagnosis was 6.0 years. In addition, 56.2% of the patients with mitochondrial nephropathy did not have elevated lactate levels.

**Table 1:** Clinical characteristics of mitochondrial nephropathy

Characteristics	Type of gene mutations					Total (*N* = 81)
	mtDNA point mutation (*n* = 66)	mtDNA single deletion (*n* = 3)	mtDNA multiple deletion (*n* = 3)	nDNA mutation related to CoQ10		
				*COQ2*/*COQ6* (*n* = 3)	*COQ8b* (*n* = 6)	
Male, *n* (%)	24 (36.4)	1 (33.3)	0 (0)	1 (33.3)	3 (50.0)	29 (35.8)
Family history, *n* (%)						
Mitochondrial disease	34 (51.5)	0 (0)	0 (0)	2 (66.7)	3 (50.0)	39 (48.2)
Diabetes	40 (60.6)	0 (0)	2 (66.7)	1 (33.3)	0 (0)	43 (53.1)
Renal manifestations during all observation periods, *n* (%)						
One of the following 3 manifestations	66 (100)	3 (100)	3 (100)	3 (100)	6 (100)	81 (100)
Decreased eGFR (<60 ml/min per 1.73 m^2^)	49 (74.2)	3 (100)	2 (66.7)	1 (33.3)	4 (66.7)	59 (72.8)
Proteinuria (≥0.15 g/g Cre)	60 (90.9)	3 (100)	3 (100)	3 (100)	6 (100)	75 (92.6)
Fanconi syndrome	3 (4.6)	3 (100)	2 (66.7)	0 (0)	0 (0)	8 (9.9)
Comorbidities during all observation periods, *n* (%)						
Diabetes	36 (54.6)	2 (66.7)	1 (33.3)	0 (0)	0 (0)	39 (48.2)
Developmental disability	25 (37.9)	3 (100)	0 (0)	2 (66.7)	0 (0)	30 (37.0)
Hearing loss	50 (75.8)	2 (66.7)	2 (66.7)	0 (0)	0 (0)	54 (66.7)
Epilepsy	15 (22.7)	1 (33.3)	1 (33.3)	1 (33.3)	0 (0)	18 (22.2)
Cardiomyopathy	18 (27.3)	2 (66.7)	0 (0)	1 (33.3)	0 (0)	21 (25.9)
No comorbidities in other organs except for kidney	4 (6.1)	0 (0)	0 (0)	0 (0)	5 (83.3)	9 (11.1)
Age at onset of renal manifestations (y.o.), median (IQR)						
One of the following 3 manifestations	20.0 (14.0–32.0)	4.0 (2.0–6.0)	39.0 (10.0–59.0)	1.0 (0–1.0)	7.5 (6.0–9.0)	19.0 (10.0–28.0)
Decreased eGFR (<60 ml/min per 1.73 m^2^)	32.5 (24.0–41.0)	—	—	—	16.5 (13.0–25.0)	32.0 (18.0–41.0)
Proteinuria (≥0.15 g/g Cre)	20.0 (13.5–28.5)	—	—	1.0 (0–1.0)	7.5 (6.0–9.0)	18.0 (9.0–27.0)
Fanconi syndrome	13.0 (3.0–14.0)	—	—	—	—	10.0 (4.0–14.0)
The onset of renal manifestations, *n* (%)						
Childhood (0–19 yr)	30 (45.4)	3 (100)	1 (33.3)	3 (100)	5 (83.3)	42 (51.9)
Adulthood (≥20 yr)	36 (54.6)	0 (0)	2 (66.7)	0 (0)	1 (16.7)	39 (48.1)
Time from renal manifestations to genetic diagnosis	6.0 (1.0–13.0)	1.0 (−4.0 to 2.0)	8.0 (6.0–23.0)	1.0 (0–1.0)	7.0 (3.0–9.0)	6.0 (1.0–12.0)
Elevation of lactate level, *n* (%)^a^	21 (42.0)	3 (100)	1 (33.3)	2 (66.7)	1 (20.0)	28 (43.8)

CoQ10, coenzyme Q10; Cre, creatinine; eGFR, estimated glomerular filtration rate; IQR, interquartile range; mtDNA, mitochondrial DNA; nDNA, nuclear DNA; y.o., years old.

a The denominator for the percentage calculation was the number of cases in which the lactate levels were measured. Elevated lactate level was defined as serum or cerebrospinal fluid lactate levels of more than 2 mmol/l (18 mg/dl) or detection of lactate peak on brain magnetic resonance spectroscopy.

### Pathologic Features of Mitochondrial Nephropathy

Among 81 patients, kidney biopsies were performed in 57 patients. The pathologic features of mitochondrial nephropathy are summarized in Table 2. Among all cases, the most common pathologic diagnosis in cases with mtDNA point mutations was FSGS (52.6%), followed by diabetic nephropathy (12.3%), and the rate of FSGS was higher in cases with coenzyme Q10-related nDNA mutations. Abnormal findings for the mitochondria, mainly in podocytes, were detected by electron microscopy in more than half of the patients who underwent renal biopsy. Granular swollen epithelial cells in the distal tubules and collecting ducts are characteristic pathologic features of mitochondrial nephropathy,^32^ but granular swollen epithelial cells were found in less than one-third of the cases in this study.

**Table 2:** Pathologic characteristics of cases with kidney biopsy

Characteristics	Type of gene mutations				Total (*N* = 57)
	mtDNA point mutation (*n* = 46)	mtDNA multiple deletion (*n* = 2)	nDNA mutation related to CoQ10		
			*COQ2*/*COQ6* (*n* = 3)	*COQ8b* (*n* = 6)	
Age at the kidney biopsy, median (IQR)	29.0 (19.0–34.0)	—	1.0 (1.0–1.0)	10.5 (7.0–19.0)	28.0 (17.0–33.0)
Data at kidney biopsy, median (IQR)					
eGFR (ml/min per 1.73 m^2^)	69.1 (44.6–97.7)	—	114.0 (19.5–170.4)	86.3 (67.3–105.3)	80.9 (46.9–107.5)
Proteinuria (g/g Cre)	1.46 (0.60–2.38)	—	50.0 (17.67–1817.6)	2.40 (1.86–4.61)	1.81 (0.90–4.20)
Pathologic diagnosis, *n* (%)					
Focal segmental glomerulosclerosis	23 (50.0)	0 (0)	2 (66.7)	5 (83.3)	30 (52.6)
Nephrosclerosis	5 (10.9)	0 (0)	0 (0)	1 (16.7)	6 (10.5)
Diabetic nephropathy	7 (15.2)	0 (0)	0 (0)	0 (0)	7 (12.3)
Tubulointerstitial nephropathy	3 (6.5)	0 (0)	0 (0)	0 (0)	3 (5.3)
Minor glomerular abnormality	3 (6.5)	2 (100)	1 (33.3)	0 (0)	6 (10.5)
Unknown	5 (10.9)	0 (0)	0 (0)	0 (0)	5 (8.8)
Abnormal findings of mitochondrial under EM, *n* (%)^a^	26 (56.5)	1 (50.0)	1 (33.3)	6 (100)	34 (59.7)
Cell type with abnormal findings of mitochondria, *n* (%)					
Podocytes	18 (39.1)	1 (50.0)	1 (33.3)	5 (83.3)	25 (43.9)
Tubular cells	16 (34.8)	1 (50.0)	0 (0)	4 (66.7)	21 (36.8)
Other (endothelial, mesangial, smooth muscle)	4 (8.7)	0 (0)	0 (0)	0 (0)	4 (7.0)
Granular swollen epithelial cells, *n* (%)	10 (21.7)	0 (0)	0 (0)	3 (50.0)	13 (22.8)

CoQ10, coenzyme Q10; Cre, creatinine; eGFR, estimated glomerular filtration rate; EM, electron microscopy; IQR, interquartile range; mtDNA, mitochondrial DNA; nDNA, nuclear DNA.

a Abnormal findings of the mitochondria are defined as an increase in the number or abnormal morphology of the mitochondria.

### Clinicopathologic Characteristics of Cases With the m.3243 A>G Mutation

As data from as many as 63 cases with the m.3243 A>G mutation were collected in this study, further analysis of these cases was attempted. In particular, we analyzed the differences between patients in whom renal manifestations occurred during childhood and those during adulthood, as these differences have been noted previously.^33^^,^^34^ The rate of cases with diabetes was significantly higher, but the rate of cases with developmental disability was significantly lower in patients with adult onset than in those with childhood onset (Table 3). Although not statistically significant, all childhood-onset patients had proteinuria, whereas 14.7% of the adult-onset patients did not have proteinuria during the observation period. Elevated lactate levels are more common in patients with childhood-onset diseases.

**Table 3:** Clinicopathologic characteristics of mitochondrial nephropathy with m.3243 A>G

Characteristics	All (*N* = 63)	Onset timing of renal manifestations^a^		
		Childhood (0–19 yr) (*n* = 28)	Adulthood (≥20 yr) (*n* = 35)	*P*
Family history, *n* (%)				
Mitochondrial disease	33 (52.4)	12 (42.9)	21 (60.0)	0.18
Diabetes	39 (61.9)	14 (50.0)	25 (71.4)	0.082
Renal manifestations during all observation periods, *n* (%)				
One of the following 3 manifestations	63 (100)	28 (100)	35 (100)	—
Decreased eGFR (<60 ml/min per 1.73 m^2^)	47 (74.6)	17 (60.7)	30 (85.7)	**0.023**
Proteinuria (≥0.15 g/g Cre)	58 (92.1)	28 (100)	30 (85.7)	0.060
Fanconi syndrome	2 (3.2)	2 (7.1)	0 (0)	0.19
Comorbidities during all observation periods, *n* (%)				
Diabetes	35 (55.6)	11 (39.3)	24 (68.6)	**0.020**
Developmental disability	23 (36.5)	14 (50.0)	9 (25.7)	**0.047**
Hearing loss	50 (79.4)	22 (78.6)	28 (80.0)	0.89
Epilepsy	13 (20.6)	9 (32.1)	4 (11.4)	0.062
Cardiomyopathy	18 (28.6)	8 (28.6)	10 (28.6)	1.00
No comorbidities in other organs except for kidney	3 (4.8)	2 (7.1)	1 (2.9)	0.58
Elevated lactate level, *n* (%)^b^	18 (38.3)	13 (65.0)	5 (18.5)	**0.001**
Cases with a kidney biopsy, *n* (%)	44 (69.8)	19 (67.9)	25 (71.4)	0.76
Data at kidney biopsy, median (IQR)				
eGFR (ml/min/1.73 m^2^)	78.8 (44.6–99.4)	95.9 (78.8–133.4)	52.0 (35.1–91.4)	**0.001**
Proteinuria (g/g Cre)	1.38 (0.60–2.00)	1.81 (0.90–3.55)	1.03 (0.50–1.97)	0.25
Pathologic diagnosis, *n* (%)				
Focal segmental glomerulosclerosis	21 (47.7)	13 (68.4)	8 (32.0)	**0.017**
Nephrosclerosis	5 (11.4)	0 (0)	5 (20.0)	0.060
Diabetic nephropathy	7 (15.9)	2 (10.5)	5 (20.0)	0.68
Tubulointerstitial nephropathy	3 (6.8)	1 (5.3)	2 (8.0)	1.00
Minor glomerular abnormality	3 (6.8)	1 (5.3)	2 (8.0)	1.00
Unknown	5 (11.4)	2 (10.5)	3 (12.0)	1.00
Abnormal findings of mitochondrial under EM, *n* (%)^c^	24 (54.6)	10 (52.6)	14 (56.0)	0.82
Cell type with abnormal findings of mitochondria, *n* (%)				
Podocytes	16 (36.4)	7 (36.8)	9 (36.0)	0.95
Tubular cells	15 (34.1)	6 (31.6)	9 (36.0)	0.76
Other (endothelial, mesangial, smooth muscle)	4 (9.1)	1 (5.3)	3 (12.0)	0.62
Granular swollen epithelial cells, *n* (%)	10 (22.7)	3 (15.8)	7 (28.0)	0.47

Cre, creatinine; eGFR, estimated glomerular filtration rate; EM, electron microscopy; IQR, interquartile range.

Bold values indicate statistically significant (*P* < 0.05).

a Time at the first detection of at least 1 of the following 3 renal manifestations was defined as the onset timing of renal manifestations: proteinuria (≥0.15 g/g Cre), reduced eGFR (<60 ml/min per 1.73 m^2^), or Fanconi syndrome.

b The denominator for the percentage calculation was the number of cases in which the lactate levels were measured. Elevated lactate level was defined as serum or cerebrospinal fluid lactate levels more than 2 mmol/l (18 mg/dl) or detection of lactate peak on brain magnetic resonance spectroscopy.

c Abnormal findings of mitochondrial are defined as an increase in the number or abnormal morphology of the mitochondria.

The rate of cases with decreased eGFR at kidney biopsy was significantly higher in adult-onset patients than in childhood-onset patients (Table 3). In addition, FSGS was the most common pathologic diagnosis in both childhood and adult-onset cases, but the rate was significantly higher in childhood-onset cases, and pathologic diagnosis in adult-onset cases was more variable, including diabetic nephropathy, nephrosclerosis, tubulointerstitial nephropathy, and even minor glomerular abnormalities.

### Prognosis of Mitochondrial Nephropathy With m.3243 A>G Depending on the Time of Onset of Renal Manifestations and Pathologic Diagnosis

The prognoses depending on differences in the time of onset of renal manifestations were also analyzed in patients who could be followed for >1 year after the onset of renal manifestations (Table 4).

**Table 4:** Prognosis of mitochondrial nephropathy with m.3243 A>G depending on the time of onset of renal manifestations

Outcomes	All (*N* = 63)	Onset timing of renal manifestations^a^		
		Childhood (0–19 yr) (*n* = 28)	Adulthood (≥20 yr) (*n* = 35)	*P*
Observation time for initiation of RRT, median (IQR) (yr)^b^	11.0 (5.0–17.0)	13.0 (7.0–18.5)	9.0 (4.0–15.0)	0.12
Initiation of RRT, *n* (%)	32 (50.8)	16 (57.1)	16 (45.7)	0.37
Observation time for all-cause mortality, median (IQR) (yr)^b^	12.0 (7.0–23.0)	14.5 (8.0–25.5)	12.0 (6.0–21.0)	0.22
All-cause mortality, *n* (%)	16 (25.4)	7 (25.0)	9 (25.7)	0.95
eGFR declining slope (ml/min per 1.73 m^2^/yr), median (IQR)	5.4 (2.9–9.2)	7.4 (4.0–10.6)	4.7 (1.7–8.2)	0.16

Cre, creatinine; eGFR, estimated glomerular filtration rate; IQR, interquartile range; RRT, renal replacement therapy.

a Time at the first detection of at least one of the following 3 renal manifestations was defined as the onset timing of renal manifestations: proteinuria (≥0.15 g/g Cre), reduced eGFR (<60 ml/min per 1.73 m^2^), or Fanconi syndrome.

b Time from onset of renal manifestations.

**Figure 2:**
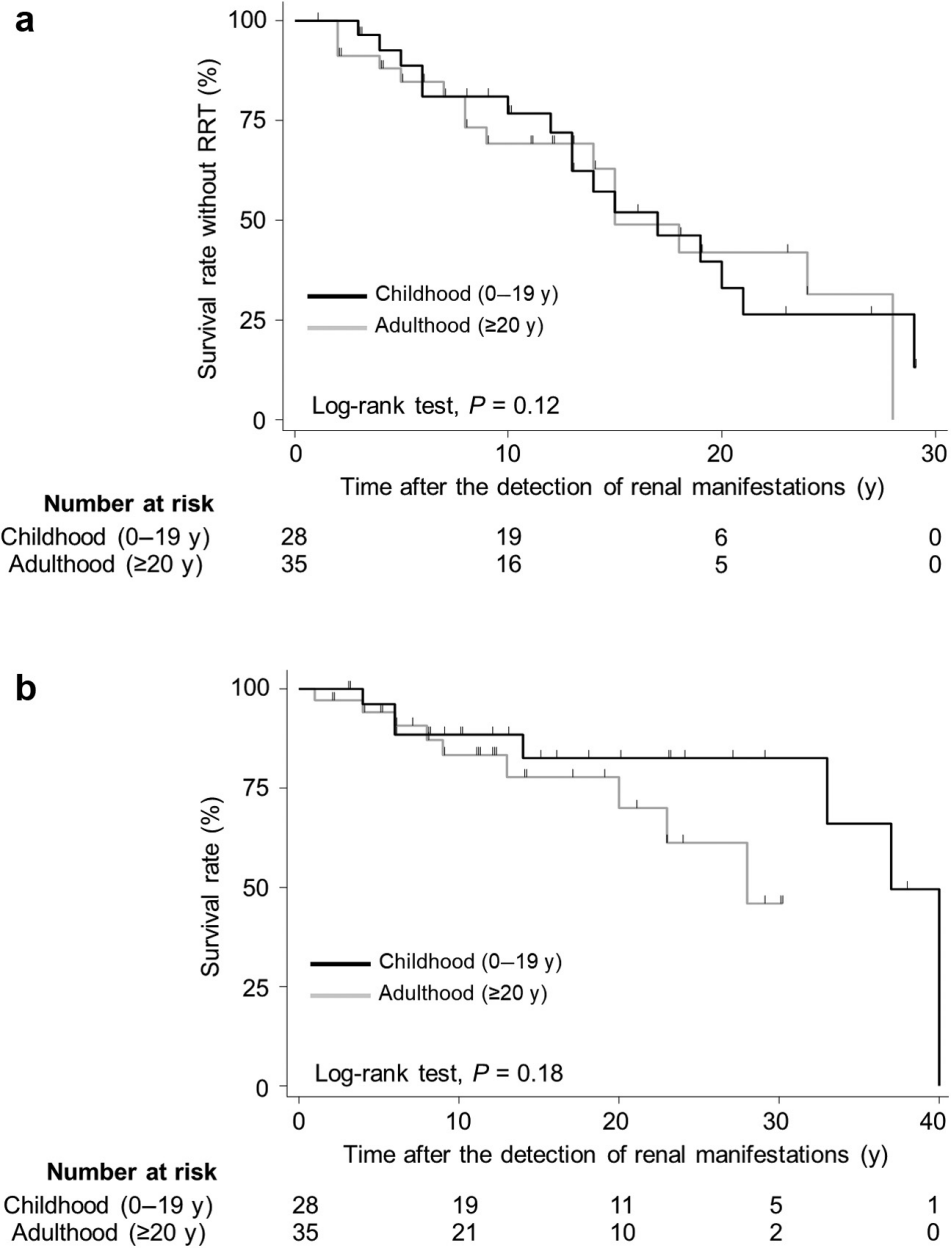
Kaplan–Meier survival (a) without RRT and (b) without death after onset of renal manifestations. The dark line indicates childhood-onset cases, and the light line indicates adult-onset cases. RRT, renal replacement therapy.

In a median follow-up period of 11.0 years after the onset of renal manifestations, RRT was initiated in 50.8% (32 of 63) of the patients (Table 4). In addition, the Kaplan–Meier analysis revealed no significant difference in the survival rate without RRT between the childhood-onset and adult-onset patients (Figure 2a). Death occurred in 25.4% (16 of 63) of the patients during a median observation period of 12.0 years after the onset of renal manifestations (Table 4). The Kaplan–Meier analysis of survival rates revealed that there was no significant difference between childhood-onset and adult-onset patients (Figure 2b). The median rate of decrease in the eGFR was 5.4 ml/min per 1.73 m^2^/yr (Table 4). The median rate of decrease in the eGFR in childhood-onset cases and adult-onset patients was 7.4 and 4.7 ml/min per 1.73 m^2^/yr, respectively, without a significant difference (*P* = 0.16) (Table 4).

Furthermore, the prognosis was analyzed according to renal pathologic diagnosis (Table 5). In FSGS cases (*n =* 21), the median rate of eGFR decline was 8.3 ml/min per 1.73 m^2^/yr, and 71.4% of the patients started RRT during the median observation period of 14.0 years and 19.1% of the patients died during the median observation period of 16.0 years. Although the number of cases analyzed was small, those with pathologic diagnosis of diabetic nephropathy by kidney biopsy (*n =* 7) had a high incidence of death (85.7%) during the median observation period of 13.0 years. In contrast, although the number of patients diagnosed with having minor glomerular abnormalities, tubulointerstitial nephropathy, and nephrosclerosis was also small (*n =* 3, 3, and 5, respectively), only 1 patient with nephrosclerosis died; excluding this case, RRT was initiated in only 1 patient with tubulointerstitial nephropathy.

**Table 5:** Clinical characteristics and prognosis of patients with m.3243 A>G by pathologic diagnosis

Characteristics of cases with mtDNA point mutation (*N* = 44)	Pathologic diagnosis		
	FSGS (*n* = 21)	Nephrosclerosis (*n* = 5)	Diabetic nephropathy (*n* = 7)
Data at kidney biopsy, median (IQR)			
Age (yr)	25.0 (18.0–30.0)	30.0 (25.0–31.0)	43.0 (33.0–61.0)
eGFR (ml/min per 1.73 m^2^)	82.3 (56.8–108.9)	44.6 (31.6–47.5)	43.9 (33.7–85.8)
Proteinuria (g/g Cre)	1.99 (1.330–4.41)	0.24 (0.04–0.97)	1.28 (0.60–2.35)
Observation time for initiation of RRT, median (IQR) (yr)^a^	14.0 (8.0–16.0)	6.0 (5.0–8.0)	13.0 (5.0–18.0)
Initiation RRT, *n* (%)	15 (71.4)	1 (20.0)	2 (42.9)
Observation time for all-cause mortality, median (IQR) (yr)^a^	16.0 (9.0–24.0)	8.0 (6.0–12.0)	13.0 (6.0–23.0)
All-cause mortality, *n* (%)	4 (19.1)	1 (20.0)	6 (85.7)
eGFR declining slope (ml/min per 1.73 m^2^/yr), median (IQR)	8.3 (4.7–13.6)	2.9 (1.7–4.5)	3.3 (0.02–16.4)

Cre, creatinine; eGFR, estimated glomerular filtration rate; FSGS, focal segmental glomerular sclerosis; IQR, interquartile range; mtDNA, mitochondrial DNA; RRT, renal replacement therapy.

a Time from onset of renal manifestations.

## Discussion

In the 1990s, there were reports of the association of renal manifestations in MDs with genetic mutations in *TI* (*tRNA*^*Ile (AUU/C)*^), *TL1* (*tRNA*^*Leu (UUR)*^), *TF* (*tRNA*^*Phe (UUU/C)*^), *TY* (*tRNA*^*Tyr (UAU/C)*^), and *TN* (*tRNA*^*Asn (AAU/C)*^) in the mtDNA.^12^, ^13^, ^14^, ^15^, ^16^, ^17^ Since 2006, studies have reported that mitochondrial nephropathy is associated with nDNA mutations related to coenzyme Q10 synthesis.^35^, ^36^, ^37^, ^38^, ^39^ In addition, mtDNA mutations in *MT-ATP6*,^40^ *MT-CO1*,^41^ and *MT-ND5*,^42^ which encode the subunits of complexes I, IV, and V, respectively, have recently been reported. Single and multiple mtDNA deletions owing to nDNA mutations can also cause mitochondrial nephropathy.^19^^,^^43^^,^^44^ With advances in genetic analysis technology and increased awareness of the disease, it is expected that the number of diagnoses of mitochondrial nephropathy will continue to increase, and new causative genes will be identified.

To date, the rate of cases with renal manifestations of MD has been studied. Massin *et al.*^45^ analyzed 74 patients with maternally inherited diabetes and deafness harboring m.3243A>G and reported that 46.3% of the patients had renal dysfunction.^45^ In contrast, Hall *et al.*^33^ analyzed 75 adult patients diagnosed with having maternally inherited diabetes and deafness, mitochondrial encephalopathy with lactic acidosis and stroke-like episodes, or myoclonic epilepsy with ragged red fibers in specialist clinics. They reported that 30.7% of the patients had albuminuria and 38.7% had elevated levels of urinary retinol-binding protein, a marker of proximal tubular damage, although none of the patients had elevated creatinine levels.^33^ These studies used different types of cohorts to extract patients with renal manifestations among MD cases. Therefore, the differences in the rate of cases with decreased renal function may vary depending on the cohort of MDs. In addition, although the clinicopathologic features of mitochondrial nephropathy with m.3243A>G have been analyzed,^22^ the data did not provide an overview of the clinical and pathologic features of mitochondrial nephropathy. In Japan, the prevalence of MDs is rare, at 2.9 per 100,000 people.^46^ Therefore, we conducted a survey on mitochondrial nephropathy by requesting the cooperation of nephrology sections throughout Japan. As a result, we collected data of 81 patients with mitochondrial nephropathy. Among them, 92.6% of the patients had proteinuria and 72.8% had decreased renal function, with gene mutations determined as the cause of MD (Table 1). We were also able to analyze 63 cases of mitochondrial nephropathy with the m.3243A>G mutation and the prognosis of these patients. This analysis could clarify not only the clinicopathologic features (Table 3) but also the prognosis (Tables 4 and 5, Figure 2) of mitochondrial nephropathy with the m.3243A>G mutation.

Nevertheless, this cohort may not accurately represent the full picture of mitochondrial nephropathy, as we conducted a survey among nephrologists. In fact, Fanconi syndrome, which is often observed in systemic MDs, accounted for a small percentage of the total population in this survey (9.9%). Furthermore, we identified only 1 case of Fanconi syndrome alone without proteinuria and decreased renal function. Therefore, we believe that this study involved a cohort of patients with mitochondrial nephropathy with proteinuria or decreased renal function, which nephrologists encounter more often. If we had included departments of metabolic diseases or emergency, Fanconi syndrome may have been more common.

The eGFR at kidney biopsy was lower in adult-onset cases than in childhood-onset cases (Table 3). A possible reason is a delay in the timing of kidney biopsy because 14.3% of adult-onset cases with the m.3243A>G mutation had no proteinuria, whereas all childhood-onset cases had proteinuria (Table 3). In addition, the median eGFR at renal biopsy in adult-onset cases with proteinuria was 59.3 (31.6–83.5) ml/min per 1.73 m^2^, whereas that in cases without proteinuria was 35.1 (33.7–44.6) ml/min per 1.73 m^2^, but the difference was not significant. These results also indicate that the “actual” onset time of mitochondrial nephropathy cannot be estimated using the definition used in this study, and it should be mentioned that cases of early onset mitochondrial nephropathy may not have been included in this study. In the future, measurement of urinary retinol-binding protein^33^ or other new biomarkers is desirable for the early detection of the onset of mitochondrial nephropathy. Furthermore, another issue to be resolved is how and when we should perform such screening in cases with a few symptoms of MDs, except for the kidney, because cases of renal-limited phenotype possibly exist in mtDNA point mutation and COQ8B mutation cases (Table 1).

The presence of complications is expected to be related to the heteroplasmy rate of mtDNA point mutations in each organ, even in the same individual, and the severity of renal disease is expected to be closely related to the heteroplasmy rate in the kidney.^5^^,^^47^ In this study, the heteroplasmy rate was not included as a survey item, and we were unable to analyze the relationship between the heteroplasmy rate and clinicopathologic features or prognosis; therefore, analyses that include heteroplasmy rate should be conducted to achieve a better understanding of the disease. The phenotypes of MDs, such as mitochondrial encephalopathy with lactic acidosis and stroke-like episodes, myoclonic epilepsy with ragged red fibers, and maternally inherited diabetes and deafness, were not included in the questions in the questionnaire, and this is another limitation of this study. Therefore, for example, although mitochondrial encephalopathy with lactic acidosis and stroke-like episodes is a well-known phenotype of MDs in cases with m.3243A>G,^4^^,^^5^ its frequency could not be evaluated in this study. Nevertheless, it is likely that most of the cases with the m.3243 A>G mutation in this study could not have been diagnosed to have mitochondrial encephalopathy with lactic acidosis and stroke-like episodes, because epilepsy complications accounted for 20% of all m.3243A>G cases (11% among adult-onset cases). In addition, we should describe the limitation of the pathologic analysis. The data for the pathologic diagnosis were not centralized and were compiled based on the pathologic data reported from each nephrology section. Furthermore, we should also consider the possibility that sampling errors of kidney biopsy specimens might have affected the results of electron microscopy data.

We included patients pathologically diagnosed with having diabetic nephropathy in the analysis of mitochondrial nephropathy. Although it has been reported that diabetes may not be a major cause of nephropathy in patients with m.3243A>G, it is important to discuss whether this prediction is justified.^33^^,^^45^ MDs account for approximately 1% of diabetes cases in Japan.^48^ In cases of diabetes caused by MD with concomitant nephropathy, it is difficult to precisely determine whether the mechanism of nephropathy in such cases is “MD → diabetes → diabetic nephropathy” or “MD → OXPHOS dysfunction in renal component cells → nephropathy.” Similar to the use of biopsied myocardial tissue to measure OXPHOS enzyme activity and diagnose MD,^49^ it would be technically feasible to measure mitochondrial enzyme activity in renal tissue using renal biopsy tissue. Nevertheless, it is difficult to obtain renal tissue for an enzyme diagnosis as a routine test in an actual clinical setting because of the risks involved. To resolve this issue, a method to evaluate the mitochondrial function of kidney cells must be developed.

The survey included patients who did not undergo genetic analyses or those who had no genetic mutations confirmed as a cause of MDs after genetic analyses. Indeed, even in cases where no genetic diagnosis was made, the data suggested pathologically increased abnormal mitochondria in podocytes, decreased enzyme activities of the mitochondrial respiratory chain in fibroblasts cultured from the skin, elevated lactate levels in the serum or cerebrospinal fluid, and cortical neurologic deficits on typical magnetic resonance imaging (Supplementary Table S2). Nevertheless, it was not possible to confirm that these patients had mitochondrial nephropathy by the definition of this study. In addition, this study revealed that the median time from onset of renal manifestations to genetic diagnosis was 6.0 years. For a better diagnosis of MDs, it is necessary to establish a diagnostic system for and create awareness of this disease. We plan to evaluate the mitochondrial function of kidney cells as a common diagnostic method for mitochondrial nephropathy.

We surveyed mitochondrial nephropathy on a wide scale to provide a complete picture of the disease and an overview of mitochondrial nephropathy. These results may lead to improvements in clinical practice and serve as a foundation for developing accurate diagnostic techniques and new treatment methods by advancing research on the pathogenesis of mitochondrial nephropathy.

## Disclosure

All the authors declared no competing interests.

## Supplementary Material

Supplementary File (PDF)

**Summary S1.** Collaborators of the J-SMiN study and collaborators responding to the questionnaire on the number of patients of mitochondrial diseases with renal manifestations.

**Summary S2.** Collaborators of the J-RBR.

**Table S1.** Rare comorbidities of mitochondrial nephropathy.

**Table S2.** Clinicopathologic features of mitochondrial nephropathy by differences in genetic analysis.
