# Supplementary material for: Clinicopathologic Features of Mitochondrial Nephropathy
Source: Kidney Int Rep. 2022 Jan 11;7(3):580–90. doi: 10.1016/j.ekir.2021.12.028 (PMC8897298; doi:10.1016/j.ekir.2021.12.028)
Supplement: Supplementary File (PDF) [file mmc1.pdf]

Supplemental Summary S1.

A list of collaborators of the J-SMiN study

Hokkaido

- Hokkaido University Hospital (Pediatrics), Takayuki Okamoto
- Hokkaido University Hospital (Nephrology), Junya Yamamoto
- National Hospital Organization Hokkaido Medical Center, Yoshinori Araki, Azusa Kawaguchi
- Teine Keijinkai Hospital, Hideki Takizawa

Iwate

- Iwate Prefectural Central Hospital, Izaya Naykaya

Miyagi

- Japan Community Health Care Organization Sendai Hospital, Toshinobu Sato

Yamagata

- Yamagata University Hospital, Kazunobu Ichikawa
- Nihonkai General Hospital, Takahiro Nakayama, Mizue Gotoh

Ibaraki

- JA Toride Medical Center, Yoshitaka Maeda
- Mito Saiseikai General Hospital, Itaru Ebihara, Chihiro Sato
- Faculty of Medicine, University of Tsukuba, Kunihiro Yamagata, Joichi Usui

Tochigi

- Jichi Medical University, Takahisa Kobayashi

Gunma

- Gunma University Graduate School of Medicine, Yasuko Kobayashi

Saitama

- Saitama Medical University, Yuko Akioka
- Saitama Children's Medical Center, Shuichiro Fujinaga, Tomohiko Nishino

Tokyo

- Kichijoji Asahi Hospital, Yoshihiro Arimura
- Toshima Hospital, Rie Okutsu, Chisato Yamamura
- The Jikei University School of Medicine (Nephrology), Takashi Yokoo, Nobuo Tsuboi
- The Jikei University School of Medicine (Pediatrics), Daishi Hirano

- The Jikei University School of Medicine (Nephrology), Hiroyuki Ueda
- Toranomon Hospital, Yoshifumi Ubara
- Toho University Faculty of Medicine, Yuko Hamasaki
- Tokyo Medical University Hachioji Medical Center, Takashi Oda, Noriko Yoshikawa
- Tokyo Metropolitan Children's Medical Center, Riku Hamada, Ryoko Harada
- Kyorin University School of Medicine, Shinya Kaname, Noriko Ikegaya
- Juntendo University Faculty of Medicine, Miyuki Takagi
- Nippon Medical School, Akiko Mii

Chiba

- The Jikei University Kashiwa Hospital, Akihiro Shimizu, Masato Ikeda
- Seirei Sakura Citizen Hospital (Pediatrics), Kazuetsu Mori, Ken Kawamura
- Seirei Sakura Citizen Hospital (Nephrology), Noriko Terasaki
- Chiba University Hospital, Masashi Aizawa
- National Hospital Organization Chibahigashi National Hospital, Toshiyuki Imasawa
- Kameda Medical Center, Tomo Suzuki

Kanagawa

- Fujisawa City Hospital, Masashi Sakai
- St. Marianna University Yokohama Seibu Hospital, Sayuri Shirai
- Yokohama City University Graduate School of Medicine, Tomohiko Kanaoka, Kouichi Tamura, Hiromichi Wakui

Nagano

- Shinshu University Hospital, Hiroki Kanazawa, Yosuke Yamada, Yuji Kamijo
- Shinshu Ueda Medical Center, Yasuhumi Takahashi
- Nagaoka Red Cross Hospital, Hajime Yamazaki
- Niigata University Medical and Dental Hospital, Takeshi Yamada

Fukui

- University of Fukui, Faculty of Medical Sciences, Naoki Takahashi, Masayuki Iwano

Shizuoka

- Shizuoka Children's Hospital, Yudai Miyama

Aichi

- Nagoya University Hospital, Shoichi Maruyama, Takuji Ishimoto
- Aichi Children's Health and Medical Center, Naoya Fujita, Satoshi Hibino

- Chubu Rousai Hospital, Yukari Murai
- Shinseikai Daiichi Hospital, Hiroshi Ogawa, Hirohumi Tamaki
- Japanese Red Cross Nagoya Daini Hospital, Yoshimitsu Gotoh

Mie

- Yokkaichi Municipal Hospital, Katsumi Ushijima

Gifu

- Gifu Prefectural General Medical Center, Eiji Matsukuma

Osaka

- Kitano Hospital, Tazuke Kofukai Medical Research Institute, Tatsuo Tsukamoto
- Yodogawa Christian Hospital, Toshiko Yoshida, Kodo Tomida
- Osaka Women's and Children's Hospital, Natsumi Yamamura

Kyoto

- Kyoto Prefectural University of Medicine, Masashi Nishida

Nara

- Nara Medical University, Kazuhiko Tsuruya, Kenichi Samejima, Kaori Tanabe

Hyogo

- Japan Community Health Care Organization Kobe Central Hospital, Takaaki Nishihara, Michitsugu Kamezaki,Yoko Adachi

Shiga

- Japanese Red Cross Otsu Hospital, Ryo Konishi, Sayako Maeda

Wakayama

- Wakayama Medical University, Yuko Shima

Okayama

- Graduate School of Medicine Dentistry and Pharmaceutical Sciences, Okayama University, Hitoshi Sugiyama, Chieko Kawakita, Masashi Kitagawa

Hiroshima

- Hiroshima University Hospital, Toshiki Doi
- National Hospital Organization Higashihiroshima Medical Center, Hiroki Tani, Taisuke Irifuku

Tokushima

- Kawashima Hospital, Yoshihiko Noma

Nagasaki

- Japan Community Health Care Organization Isahaya General Hospital, Hideyuki Arai

Saga

- Saga University, Faculty of Medicine, Yasufumi Ohtsuka

Fukuoka

- National Hospital Organization Kyushu Medical Center, Masaru Nakayama
- Fukuoka Children’s Hospital, Yoshitsugu Kaku
- Kurume University Hospital, Yusuke Kaida, Kei Fukami

Miyazaki

- University of Miyazaki Hospital (Pediatrics), Hideaki Imamura
- University of Miyazaki Hospital (Nephrology), Shouichi Fujimoto

Kumamoto

- Faculty of Life Sciences Kumamoto University, Hiroko Nagata

Kagoshima

- Kagoshima University Hospital, Kouki Tokunaga, Haruhito Yoshimine

Okinawa

- Nanbu Medical Center & Nanbu Children's Medical Center, Tomoo Kise

<u>A list of collaborators responding to the questionnaire on the number of patients of mitochondrial diseases with renal manifestations</u>

Hokkaido

- Teine Keijinkai Hospital, Hideki Takizawa
- Kin-ikyo Chuo Hospital, Satoko Iriuda
- Oji General Hospital, Yukiyasu Fujise, Takahito Itoh
- Sapporo Medical University Hospital, Norihito Moniwa
- Sapporo City General Hospital, Yuichiro Fukazawa, Takahiro Tsuji
- Hokkaido University Hospital (Pediatrics), Takayuki Okamoto
- Hokkaido University Hospital (Nephrology), Junya Yamamoto
- National Hospital Organization Hokkaido Medical Center (Nephrology), Sekiya Shibazaki
- National Hospital Organization Hokkaido Medical Center (Pediatric nephrology), Yoshinori Araki, Azusa Kawaguchi
- Aiiku Hospital, Satoshi Sasaki

- Japanese Red Cross Asahikawa Hospital, Hironori Kobayashi
- Asahikawa Medical University, Naoki Nakagawa
- Kushiro Red Cross Hospital, Minoru Makita
- Japanese Red Cross Kitami Hospital, Koki Shimada

Aomori

- National Hospital Organization Hirosaki National Hospital (Pediartics), Kazushi Tsuruga
- Hirosaki University Hospital, Hiroshi Tanaka, Koji Tsugawa

Iwate

- Iwate Prefectural Central Hospital, Izaya Naykaya
- Iwate Medical University Hospital, Koichi Asahi

Miyagi

- Tohoku Kosai Hospital, Kastuya Obara
- Tohoku University School of Medicine (Pediatrics), Noriko Sugawara
- Tohoku University School of Medicine (Nephrology), Mariko Miyazaki
- Japan Community Health Care Organization Sendai Hospital, Toshinobu Sato
- Tohoku Medical and Pharmaceutical University, Tetsuji Morimoto

Akita

- Akita University Hospital, Atsushi Komatsuda
- Akita Kouseiren, Hiroshi Ohtani

Yamagata

- Yamagata City Hospital Saiseikan, Noriyuki Degawa
- Yamagata University Faculty of Medicine (Pediatrics), Daisuke Ogino
- Yamagata University Hospital, Kazunobu Ichikawa
- Nihonkai General Hospital, Takahiro Nakayama, Mizue Gotoh

Fukushima

- Fukushima Medical University (Pediatrics), Yukihiko Kawasaki, Kazuhide Suyama
- Fukushima Medical University (Nephrology), Junichiro Kazama
- Tokiwa Group Jyoban Hospital, Hiroshi Kawaguchi
- Fukushima Rosai Hospital, Kusano Yuki

Ibaraki

- Kandatsu Hospital, Hiroyuki Otani
- Tokyo University Ibaraki Medical Center, Masaki Kobayashi
- Tsukuba Central Hospital, Takashi Ishizu

- Medical Corp.JONINKAI Ushiku Aiwa General Hospital, Hideko Nakamura
- JA Toride Medical Center, Yoshitaka Maeda
- Faculty of Medicine, University of Tsukuba, Kunihiro Yamagata, Joichi Usui
- Koga Red Cross Hospital, Sumiko Honma
- Mito Saiseikai General Hospital, Itaru Ebihara, Chihiro Sato
- Kamisu Saiseikai Hospital, Kei Nagai
- Hitachi General Hospital, Atsushi Ueda

Tochigi

- Shin-Oyama City Hospital, Takahisa Kobayashi
- Japanese Red Cross Ashikaga Hospital, Keita Hirano
- Sano Kosei General Hospital, Marohito Murakami
- Jichi Medical University, Takahisa Kobayashi
- International University of Health and welfare Hospital, Naoki Washida

Gunma

- Hidaka-kai Hidaka Hospital, Takaaki Tsutsui
- Gunma Saiseikai Maebashi Hospital, Keiichiro Mishima
- Gunma University Graduate School of Medicine (Nephrology), Keiju Hiromura
- Gunma University Graduate School of Medicine (Pediatrics), Yasuko Kobayashi
- Shibukawa Central Hospital, Tokuyuki Kitahara

Saitama

- Saitama Medical Center Jichi Medical University, Yoshiyuki Morishita
- Saitama Children's Medical Center, Shuichiro Fujinaga, Tomohiko Nishino
- JCHO Saitama Northern Center, Yutaka Kuroda
- Saiyu Soka Hospital, Isao oosawa
- Soka Municipal Hospital, Shin Suda
- Misato Kenwa Hospital, Kimihiko Matsuyama
- Dokkyo Medical University Saitama Medical Center, Tetsuro Takeda
- Shuuwa General Hospital, Satomi Shikuma
- Shiraoka Central General Hospital, Masao Sato
- Saitama Medical University (Nephrology), Koichi Okada
- Saitama Medical University (Pediatrics), Yuko Akioka
- Saitama Sekishinkai Hospital, Shiko Gen
- Gyoda General Hospital, Takahiko Sato
- Ageo Central General Hospital, Kenichiro Kojima

Chiba

- Chiba University Hospital, Katsuhiko Asanuma, Masashi Aizawa
- National Hospital Organization Chibahigashi National Hospital (Pediatrics), Chieko Matsumura
- National Hospital Organization Chibahigashi National Hospital (Nephrology), Toshiyuki Imasawa
- Chiba Children's Hospital, Masataka Hisano
- Matsudo City General Hospital, Shinsuke Matsumoto, Masato Mori
- The Jikei University Kashiwa Hospital, Akihiro Shimizu, Masato Ikeda
- Juntendo University Urayasu Hospital, Naoto Nishizaki, Taichi Hara
- Seirei Sakura Citizen Hospital (Pediatrics), Kazuetsu Mori, Ken Kawamura
- Seirei Sakura Citizen Hospital (Nephrology), Satoshi suzuki, Takayuki Fujii, Noriko Terasaki
- Japanese Red Cross Narita Hospital, Hirofumi Ohashi, Nobuhiko Kuramoto
- Kameda Medical Center, Mamiko Ohara, Tomo Suzuki

Tokyo

- Nihon University Hospital, Shinsuke Harasawa
- Mitsui Memorial Hospital, Naobumi Mise
- Luke's International Hospital, Masaaki Nakayama
- The Jikei University School of Medicine (Pediatrics), Daishi Hirano
- The Jikei University School of Medicine (Nephrology), Takashi Yokoo, Nobuo Tsuboi
- Toranomon Hospital, Tetsushi Ogawa
- Juntendo University Faculty of Medicine, Miyuki Takagi
- Nippon Medical School, Akiko Mii
- The University of Tokyo Hospital, Yoshifumi Hamasaki, Masaomi Nangaku
- Tokyo-Kita Medical Center, Yaeko Motoyoshi
- The Jikei University Katsushika Medical Center, Shinya Yokote
- Tokyo Metropolitan Bokutoh Hospital, Tae Omori
- Koto Hospital, Isao Ebihara, Yuichi Tanaka, Sei Akatsuka
- Showa University School of Medicine, Yoshifusa Abe
- Tokyo Rosai Hospital, Takashi Naitou
- Omori Red Cross Hospital, Ken Shibuya
- Toho University Faculty of Medicine, Yuko Hamasaki
- National Hospital Organization Tokyo Medical Center, Tomokazu Matsuura
- Toho University Ohashi Medical Center, Nobuhiko Joki, Toshihide Hayashi
- Tokyo Kyosai Hospital, katsuyuki Ooi

- National Center for Child Health and Development, Koichi Kamei
- Nissan Tamagawa Hospital, Yoshihiko Imamura
- Tokyo Medical University Hospital, Yoshihiko Kanno, Yume Nagaoka
- Ohkubo Hospital, Sachiko Wakai
- Keio University Hospital, Hiroshi Itoh, Kazutoshi Miyashita
- Tokyo Women's Medical University, Motoshi Hattori, Kenichiro Miura
- Tokyo Metropolitan Police Hospital, Tomonari Okada
- Nitobe Memorial Nakano General Hospital, Yumi Noda
- Kawakita General Hospital, Takahiro Okai
- Tokyo Metropolitan Ohtsuka Hospital, Momono Yoshikawa
- "Tokyo Metropolitan Health and Medical Corporation
- Toshima Hospital", Rie Okutsu
- Teikyo University, Yoshihide Fujigaki
- Itabashi Chuo Medical Center, Yusuke Tsukamoto
- Juntendo University Nerima Hospital, Yoshiyuki Ootomo
- Kichijoji Asahi Hospital, Takashi Yasuda, Yoshihiro Arimura
- Kyorin University School of Medicine (Pediatrics), Eriko Tanaka
- Kyorin University School of Medicine (Nephrology), Shinya Kaname, Noriko Ikegaya
- Tokyo Metropolitan Children's Medical Center, Riku Hamada, Ryoko Harada
- Musashino Tokusukai Hospital, Hiromichi Suzuki
- Tachikawa Sogo Hospital, Hiroshi Koizumi, Soh Suzuki
- Tokai University Hachioji Hospital, Shoujirou Okamoto
- Tokyo Medical University Hachioji Medical Center, Takashi Oda, Noriko Yoshikawa
- Tokyo-Nishi Tokushukai Hospital, Kyoko Maesato
- The Jikei University School of Medicine (Nephrology), Hiroyuki Ueda
- Nippon Medical School Tama Nagayama Hospital, Tomohiro Kaneko

Kanagawa

- Kawasakisaiwai Hospital, Susumu Uda
- Toranomon Hospital Kajigaya, Yoshifumi Ubara
- St. Marianna University School of Medicine, Yugo Shibagaki, Daisuke Ichikawa
- Yokohama City University Graduate School of Medicine (Pediatrics), Shuichi Ito
- Yokohama City University Graduate School of Medicine (Nephrology), Tomohiko Kanaoka, Kouichi Tamura, Hiromichi Wakui
- Seirei Yokohama Hospital, Satoshi Hirade

- St. Marianna University Yokohama Seibu Hospital, Sayuri Shirai
- National Hospital Organization Yokohama Medical Center, Kei Matsushita
- Shonan Kamakura General Hospital, Shuzo Kobayashi, Takayasu Ohtake
- Shonan Fujisawa Tokushukai Hospital, Machiko Oka
- Fujisawa City Hospital, Masashi Sakai
- Kitasato University, Kenji Ishikura, Shinya Kon
- Hiratsuka City Hospital, Toshio Imafuku
- Japanese Red Cross Hadano Hospital, Toshikazu Takizawa
- Tokai University Hospital (Pediatrics), Fumio Niimura
- Tokai University Hospital (Nephrology), Takehiko Wada

Yamanashi

- Haraguchi Clinic, Kazutaka Haraguchi
- University of Yamanashi Hospital, Kenichiro Kitamura, Fumihiko Furuya

Nagano

- Nagano Red Cross Hospital, Mamoru Kobayashi
- Shinshu Ueda Medical Center, Yasuhumi Takahashi
- Kanno Dialysis & Vascular Access Clinic, Yutaka Kanno, Taro Kanno
- Aizawa Hospital, Tomomasa Oguchi
- Shinshu University Hospital, Hiroki Kanazawa, Yosuke Yamada, Yuji Kamijo
- Ina Central Hospital, Hiroaki Takemae
- Matsumoto Medical Center, Satoshi Fujita

Niigata

- Koyo Takata Clinic, Takuma Takata
- Nagaoka Red Cross Hospital, Hajime Yamazaki
- Niigata Prefectural Central Hospital, Fumihiro Akiyama
- JA Niigata Kouseiren Ojiya General Hospital, Ryouta Yasukawa
- Minamiuonuma City Hospital, Kaoru Tabei
- Uonuma Kikan Hospital, Noriaki Iino
- Niigata Rinko Hospital, Yutajaka Osawa
- Kido Hospital, Hitomi Hama
- Saiseikai Niigata Hospital, Kazuyuki Tasaki
- Niigata University School of Medicine (Nephrology), Ichiei Narita, Yumi Ito
- Niigata University Medical and Dental Hospital, Takeshi Yamada
- Sanjo General Hospital, Yoichi Iwafuchi

Toyama

- Toyama University Hospital, Hidenori Yamazaki
- Toyama Red Cross Hospital, Takashi Kawane
- Toyama Prefectural Central Hospital, Masahiko Kawabata
- Saiseikai Toyama Hospital, Michio Matsumoto
- Kanazawa Medical University Himi Municipal Hospital, Takuma Fujiki
- Kurobe City Hospital, Shinichi Takeda

Ishikawa

- Kanazawa Nishi Hospital, Tetsuji Hashiba
- Kanazawa Medical University, Kengo Furuichi, Hitoshi Yokoyama
- Koshino Internal Medicine Clinic, Yoshitaka Koshino
- Ishikawa Prefectural Central Hospital, Akikatsu Nakashima
- Asanogawa General Hospital, Hiroshi Okuyama
- Kanazawa University Hospital (Pediatrics), Masaki Shimizu
- Kanazawa University Hospital (Nephrology), Takashi Wada, Taro Miyagawa
- National Hospital Organization Kanazawa Medical Center (Nephrology), Kiyoki Kitagawa
- National Hospital Organization Kanazawa Medical Center (Pediatrics), Kazuhide Ohta
- Kaga Medical Center, Kazuaki Mizutomi
- Komatsu Municipal Hospital, Hisashi Kaneda
- Public Central Hospital of Matto Ishikawa, Chikako Takaeda
- Keiju Medical Center, Masahide Yamazaki, Kiyotaka Mukai

Fukui

- University of Fukui, Faculty of Medical Sciences, Naoki Takahashi, Masayuki Iwano
- Japan Community Health Care Organization Fukui Katsuyama General Hospital, Yukiko Mori
- Sugita Genpaku Memorial Obama Municipal Hospital, Haruyoshi Yoshida
- Japanese Red Cross Fukui Hospital, Soichi Tamamura

Shizuoka

- Fuji City General Hospital, Kenji Kasai
- Shizuoka Municipal Hospital, Yoshihiro Matsumoto
- Shizuoka Children's Hospital, Yudai Miyama
- Yaizu City Hospital, George Seki
- Seirei Hamamatsu General Hospital (Nephrology), Taro Misaki

- Seirei Hamamatsu General Hospital (Pediatrics), Masaki Yamamoto
- Hamamatsu University School of Medicine, Hideo Yasuda
- Iwata City Hospital, Hirotaka Fukasawa

Aichi

- Okazaki City Hospital, Hiroaki Asada
- Chubu Rousai Hospital, Yukari Murai
- National Hospital Organization Nagoya Medical Center, Tomonobu Nakamura
- Nagoya City University West Medical Center, Norihiro Suga
- Nagoya City University East Medical Center, Tatsuya Tomonari
- Nagoya University Hospital, Shoichi Maruyama, Takuji Ishimoto
- Japanese Red Cross Nagoya Daini Hospital (Nephrology), Asami Takeda
- Japanese Red Cross Nagoya Daini Hospital (Pediatric Nephrology), Yoshimitsu Gotoh
- Shinseikai Daiichi Hospital, Hiroshi Ogawa, Hirohumi Tamaki
- Fujita Health University (Pediatrics), Yohei Ikezumi
- Fujita Health University (Nephrology), Yukio Yuzawa, Hiroki Hayashi
- Aichi Children's Health and Medical Center, Naoya Fujita, Satoshi Hibino
- Handa City Hospital, Makoto Mizutani
- Aichi Medical University, Yasuhiko Ito, Hironobu Nobata
- Kasugai Municipal Hospital, Tomohiko Naruse
- Tosei General Hospital, Shinichiro Inaba

Gifu

- Gifu Prefectural General Medical Center, Eiji Matsukuma

Mie

- Japan Community Health Care Organization Yokkaichi Hazu Medical Center, Yasuhide Mizutani, Masato Miyake
- Yokkaichi Municipal Hospital (Nephrology), Tomohiro Masuda
- Yokkaichi Municipal Hospital (Pediatrics), Katsumi Ushijima
- Suzuka Kaisei Hospital, Koji Matsuo

Osaka

- Kitano Hospital, Tazuke Kofukai Medical Research Institute, Tatsuo Tsukamoto
- Yodogawa Christian Hospital, Toshiko Yoshida, Kodo Tomida
- Osaka City General Hospital (Nephrology), Takashi Morikawa
- Osaka City General Hospital (Pediatrics), Rika Fujimaru
- National Hospital Organization Osaka National Hospital, Hirotsugu Iwatani

- Otemae Hospital, Toshihiro Sugiura
- Osaka Saiseikai Izuo Hospital, Yasukiyo Mori
- Kansai Electric Power Hospital, Toshiyuki Komiya
- Minamiosaka Hospital, Yasuro Kumeda
- Toyonaka Municipal Hospital, Masanobu Takeji
- Inoue Hospital, Yoshihiro Tsujimoto, Yuko Nonaka
- Osaka University Graduate School of Medicine, Yoshitaka Isaka
- Osaka Medical and Pharmaceutical University Hospital, Tatsuhiko Mori
- Kansai Medical University Hospital, Takahisa Kimata
- Arisawa General Hospital, Hyogo Nakakura
- Ishikiriseiki Hospital, Msahito Imanishi
- Ishikai Yao General Hospital, Kaname Hirowatari
- Kindai University Faculy of Medicine, Keisuke Sugimoto
- Sakai Chikamori Hospital, Norio Komai
- Osaka Women's and Children's Hospital, Natsumi Yamamura

Hyogo

- Hara Hospital, Kunihiko Yoshiya
- Kobe University Graduate School of Medicine, Kandai Nozu
- Hyogo Prefectural Kobe Children's Hospital, Ryojirou Tanaka
- Japan Community Health Care Organization Kobe Central Hospital, Takaaki Nishihara, Michitsugu Kamezaki,Yoko Adachi
- Kobe City Nishi-Kobe Medical Center, Kazuo Torigoe
- Konan Medical Canter, Akira Fujimori
- Hyogo College Of Medicine, Takayuki Shibano, Yasuhiko Tanaka
- Shiso Municipal Hospital, Yuki Yamashiro
- AIJINKAI Healthcare Corporation Akashi Medical Center, Yuriko Yonekura
- Kitaharima medical center, Natsuki Matsunoshita
- Kakogawa City Hospital, Atsushi Shirai

Nara

- Nara Prefecture General Medical Center, Masaru Matsui
- Takanohara Central Hospital, Hitomi Kawano
- Nara Medical University, Kazuhiko Tsuruya, Kenichi Samejima, Kaori Tanabe
- Nara Prefecture Western Medical Center, Katsuhiko Morimoto

Kyoto

- Kyoto Prefectural University of Medicine (Nephrology), Keiichi Tamagaki

- Kyoto Prefectural University of Medicine, Masashi Nishida
- Kyoto University Hospital, Kaoru Sakai
- Rakuwakai Otowa Hospital, Takahiko Nakagawa
- Tojinkai Hospital, Toshiko Tokoro
- National Hospital Organization Kyoto Medical Center, Koichi Seta, Kensei Yahata
- Mitsubishi Kyoto Hospital, Satoshi Matsui

Shiga

- Otsu City Hospital, Jun Nakazawa
- Shiga University of Medical Science, Toshihiro Sawai
- Saiseikai Shigaken Hospital, Tetsuya Makiishi
- Japanese Red Cross Otsu Hospital, Ryo Konishi, Sayako Maeda

Wakayama

- Japanese Red Cross Wakayama Medical Center, Yoshihito Higashi
- Wakayama Medical University, Yuko Shima
- Kinan Hospital, Seiji Hashimoto

Tottori

- Faculty of Medicine Tottori University, Shinichi Okada
- Tottori University Hospital, Chishio Munemura, Satoko Fukuda

Shimane

- Matsue Red Cross Hospital, Yoshinori Urushidani
- Matsue Seikyo General Hospital, Manabu Shiono
- Shimane Prefectural Central Hospital, Yasuo Itoh, Kosuke Matsui

Okayama

- Okayama Saiseikai General Hospital, Takayuki Miyai
- Okayama City Hospital, Keiichi Takiue
- Okayama University (Pediatrics), Hirokazu Tsukahara, Hiroyuki Miyahara
- Graduate School of Medicine Dentistry and Pharmaceutical Sciences, Okayama University, Hitoshi Sugiyama, Chieko Kawakita, Masashi Kitagawa
- Kawasaki Medical School, Tamaki Sasaki
- Shigei Medical Research Hospital, Masaki Fukushima
- National Hospital Organization Okayama Medical Center (Nephrology), Kosuke Ota
- National Hospital Organization Okayama Medical Center (Pediatrics), Junya Shimizu
- Kurashiki Central Hospital, Kenichiro Asano

- Mizushima Kyodo Hospital, Nobuyoshi Sugiyama

Hiroshima

- Shobara Redcross Hospital, Koichiro Nakashima, Yumi Honda
- Hiroshima Red Cross Hospital & Atomic-bomb Survivors Hospital, Yukio Yokoyama
- Hiroshima University Hospital, Takao Masaki, Toshiki Doi
- National Hospital Organization Higashihiroshima Medical Center, Taisuke Irifuku

Yamaguchi

- Shimonoseki City Hospital, Shoji Sakai

Tokushima

- Tokushima University (Pediatrics), Shoji Kagami, Maki Urushihara
- Tokushima University (Nephrology), Kojiro Nagai
- Kawashima Hospital, Yoshihiko Noma
- Tokushima Red Cross Hospital, Motokazu Matsuura

Kagawa

- Kinashi Obayashi Hospital, Makoto Kinashi, Hiroaki Obayashi
- Shikoku Medical Center for Children and Adults, Shuji Kondo

Kochi

- Kochi Medical School (Nephrology), Yoshio Terada
- Kochi Medical School (Pediatrics), Masayuki Ishihara

Fukuoka

- Saiseikai Yahata General Hospital, Chikao Yasunaga
- National Hospital Organization Kyushu Medical Center, Masaru Nakayama
- Harasanshin Hospital, Hideki Yotsueda
- Fukuoka City Hospital, Hiroshi Ikeda
- Fukuoka Children’s Hospital, Yoshitsugu Kaku
- Japanese Red Cross Fukuoka Hospital (Pediatrics), Ken Hatae
- Japanese Red Cross Fukuoka Hospital (Nephrology), Koji Mitsuiki, Kentaro Nakai
- Iizuka Memorial Hospital, Kazuhito Takeda
- Kurume University Hospital, Yusuke Kaida,Kei Fukami
- Yame General Hospital, Ohara Atsuko
- Omuta City Hospital, Masahiro Tanaka
- Koga Hospital 21, Seiya Okuda

Saga

- National Hospital Organization Ureshino Medical Center, Tsuyoshi Takashima
- Saga University, Faculty of Medicine (Nephrology), Yuji Ikeda
- Saga University, Faculty of Medicine, Yasufumi Ohtsuka

Nagasaki

- Saiseikai Nagasaki Hospital, Atsushi Mori
- Nagasaki University Hospital, Tomoya Nishino, Tadashi Uramatsu
- Japan Community Health Care Organization Isahaya General Hospital, Hideyuki Arai
- National Hospital Organization Nagasaki Medical Center, Akihiro Maekawa
- Sasebo Kyosai Hospital, Ken-ichi Fukunari

Kumamoto

- National Hospital Organization Kumamoto Medical Center, Masao Tomita
- Kumamoto University Hospital, Masataka Adachi, Yutaka Kakizoe
- Faculty of Life Sciences Kumamoto University, Hiroko Nagata
- Kumamoto Red Cross Hospital, Souichi Uekihara, Mariko Toyoda
- Kumamoto Chuo Hospital, Kenji Arizono, Kazufumi Nomura
- Japan Community Health Care Organization Kumamoto General Hospital, Taku Miyoshi

Oita

- Oita Red Cross Hospital, Kouji Kaneda
- JA Oita Koseiren Tsurumi Hospital , Ryokichi Yasumori

Miyazaki

- Miyazaki Prefectural Miyazaki Hospital, Naoko Ikeda
- Faculty of Medicine, University of Miyazaki, Hideaki Imamura
- University of Miyazaki Hospital, Shouichi Fujimoto

Kagoshima

- Jiaikai Imamura General Hospital, Miki Uwatoko,Mayumi Fukumoto
- Kagoshima University Hospital, Akio Ido, Koki Tokunaga

Okinawa

- Nanbu Medical Center & Children's Medical Center, Tomoo Kise
- University of the Ryukyus Hospital (Pediatrics), Koichi Nakanishi, Sadao Nakamura
- University of the Ryukyus Hospital (Nephrology), Kentaro Kohagura, Takan

Supplemental Summary S2.

A list of collaborators of the J-RBR

Appendix

The following investigators and initial institutions have participated in the development of the J-RBR since 2007: Hirofumi Makino and Hitoshi Sugiyama (Okayama University), *late* Takashi Taguchi (Nagasaki University), Hitoshi Yokoyama (Kanazawa Medical University), Hiroshi Sato (Tohoku University; present institution: JR Sendai Hospital), Takao Saito (Fukuoka University; present institution: Sanko Clinic), Yoshie Sasatomi (Fukuoka University; present institution: Kanenokuma Hospital), Yukimasa Kohda (Kumamoto University; present institution: Hikarinomori Clinic), Shinichi Nishi (Niigata University; present institution: Kobe University), Kazuhiko Tsuruya (Kyushu University; present institution: Nara Medical University), Yutaka Kiyohara (Kyushu University; present institution: Hisayama Research Institute for Lifestyle Diseases), Hideyasu Kiyomoto (Kagawa University; present institution: Tohoku Medical Megabank Organization, Tohoku University), Hiroyuki Iida (Toyama Prefectural Central Hospital; present institution: Toyama Prefectural Rehabilitation Hospital), Tamaki Sasaki (Kawasaki Medical School),*late* Makoto Higuchi (Shinshu University), Motoshi Hattori (Tokyo Women's Medical University), Kazumasa Oka (Osaka Kaisei Hospital; present institution: Hyogo Prefectural Nishinomiya Hospital), Shoji Kagami (The University of Tokushima Graduate School), Michio Nagata (University of Tsukuba), Tetsuya Kawamura (The Jikei University School of Medicine), Masataka Honda (Tokyo Metropolitan Children's Medical Center), Yuichiro Fukasawa (KKR Sapporo Medical Center; present institution: Sapporo City General Hospital), Atsushi Fukatsu (Kyoto University Graduate School of Medicine; present institution: Fukatsu Medical Clinic), Kunio Morozumi (Japanese Red Cross Nagoya Daini Hospital; present institution: Masuko Memorial Hospital), Norishige Yoshikawa (Wakayama Medical University; present institution: Takatsuki General Hospital), Yukio Yuzawa (Fujita Health University), Seiichi Matsuo (Nagoya University) and Kensuke Joh (Chiba-East National Hospital; present institution: The Jikei University School of Medicine).

Hokkaido District

・Asahikawa Medical University Hospital (Division of Cardiology, Nephrology, Pulmonology and Neurology, Department of Internal Medicine), Naoyuki Hasebe, Naoki Nakagawa

・National Hospital Organization Hokkaido Medical Center (Department of Nephrology), Sekiya Shibazaki, Tomotsune Miyamoto, Masanori Ito

・Hokkaido University Graduate School of Medicine (Department of Rheumatology, Endocrinology and Nephrology, Faculty of Medicine and Graduate School of Medicine, Hokkaido University), Saori Nishio, Daigo Nakazawa

・Hokkaido University Graduate School of Medicine (Department of Pediatrics), Takayuki Okamoto, Yasuyuki Sato

・KKR Sapporo Medical Center (Department of Pathology), Akira Suzuki

- Sapporo Medical University (Department of Cardiovascular, Renal and Metabolic Medicine), Norihito Moniwa, Marenao Tanaka
- Sapporo City General Hospital, Yuichiro Fukasawa
- Teine Keijinkai Hospital (Department of Nephrology), Hideki Takizawa

Tohoku District

- Iwate Prefectural Central Hospital (Department of Nephrology and Rheumatology), Jun Soma, Izaya Nakaya
- Fukushima Medical University (Department of Nephrology and Hypertension), Junichiro James Kazama, Kenichi Tanaka, Mizuko Tanaka
- Japan Community Health care Organization Sendai Hospital (Department of Nephrology), Toshinobu Sato, Satoru Sanada, Hideaki Hashimoto
- Tohoku University Hospital and affiliated hospitals (Internal Medicine), Mariko Miyazaki, Tasuku Nagasawa, Koji Okamoto
- Yamagata University School of Medicine (Department of Cardiology, Pulmonology, and Nephrology), Tsuneo Konta, Kazunobu Ichikawa
- Yamagata University School of Medicine (Department of Pediatrics), Daisuke Ogino

Kanto District

- National Hospital Organization Chibahigashi National Hospital (Department of Pathology), Hiroshi Kitamura, (Department of Nephrology), Toshiyuki Imasawa, (Department of Pediatrics), Chieko Matsumura, (Department of Surgery), Naotake Akutsu
- National Hospital Organization Chiba-East Hospital (Department of Urology), Koichi Kamura (*) *present address, Harunclinic Sakura
- Dokkyo Medical University Saitama Medical Center (Department of Nephrology), Tetsuro Takeda
- Dokkyo Medical University (Department of Nephrology and Hypertension), Toshihiko Ishimitsu
- Gunma University Graduate School of Medicine (Department of Nephrology and Rheumatology), Keiju Hiromura, Yoriaki Kaneko, Hidekazu Ikeuchi, Toru Sakairi
- Jichi Medical University (Division of Nephrology), Daisuke Nagata, Osamu Saito, Tetsu Akimoto
- Jichi Medical University, Saitama Medical Center (Division of Nephrology, Department of Integrated Medicine), Yoshiyuki Morishita
- The Jikei University School of Medicine (Division of Nephrology and Hypertension) Takashi Yokoo, Nobuo Tsuboi, Hiroyuki Ueda, Kentaro Koike, Go Kanzaki
- The Jikei University School of Medicine, Katsushika Medical Center (Division of Nephrology and Hypertension), Yudo Tanno, Shinya Yokote
- The Jikei University School of Medicine, Daisan Hospital (Division of Nephrology and Hypertension), Yoichi Miyazaki, Masahiro Okabe
- The Jikei University Kashiwa Hospital (Division of Nephrology and Hypertension), Masato Ikeda, Akihiro Shimizu

- Juntendo University Faculty of Medicine (Department of Nephrology), Yusuke Suzuki, Tomohito Goda, Masao Kihara, Miyuki Takagi
- Japanese Red Cross Ashikaga Hospital (Department of Nephrology), Keita Hirano
- Kawaguchi Municipal Medical Center (Division of Nephrology), Masahiro Ishikawa
- Kyorin University School of Medicine (Department of Nephrology and Rheumatology), Shinya Kaname, Kazuhito Fukuoka, Takahisa Kawakami
- Mito Saiseikai General Hospital (Division of Nephrology), Itaru Ebihara, Chihiro Sato
- Nippon Medical School (Division of Nephrology, Department of Internal Medicine), Yukinao Sakai, Akio Hirama, Akiko Mii
- Nihon University School of Medicine (Division of Nephrology, Hypertension and Endocrinology), Yoshinobu Fuke, Masanori Abe
- Saitama Medical University, Faculty of Medicine (Department of Nephrology), Hirokazu Okada, Tsutomu Inoue
- Saitama Medical University, Saitama Medical Center (Department of Nephrology and Hypertension), Takatsugu Iwashita, Akito Maeshima, Hajime Hasegawa
- Saiyu Soka Hospital （Department of Internal Medicine), Masamitsu Ubukata
- Showa University School of Medicine (Division of Nephrology, Department of Medicine), Masayuki Iyoda, Takanori Shibata
- Showa University Fujigaoka Hospital (Division of Nephrology, Department of Medicine), Yoshihiko Inoue
- St. Marianna University School of Medicine (Division of Nephrology and Hypertension, Department of Internal Medicine), Tomo Suzuki, Daisuke Ichikawa, Sayuri Shirai, Yugo Shibagaki
- Tokai University School of Medicine (Division of Nephrology, Endocrinology and Metabolism), Takehiko Wada, Masafumi Fukagawa
- Teikyo University School of Medicine (Department of Internal Medicine), Yoshihide Fujigaki
- Teikyo University School of Medicine (Department of Urology), Shigeo Horie(*), Satoru Muto(*) *present address, Juntendo University School of Medicine (Department of Urology)
- Tokyo Medical University Ibaraki Medical Center (Department of Nephrology), Masaki Kobayashi, Kouichi Hirayama, Homare Shimohata
- Tokyo Metropolitan Children's Medical Center (Department of Nephrology), Riku Hamada (Department of General Pediatrics), Hiroshi Hataya
- Tokyo Women's Medical University (Department of Pediatric Nephrology), Motoshi Hattori, Kenichiro Miura, Kiyonobu Ishizuka, Naoto Kaneko
- Tokyo Women's Medical University (Department of Nephrology), Kosaku Nitta, Keiko Uchida, Takahito Moriyama
- Toranomon Hospital, Nephrology Center, Yoshifumi Ubara, Tatsuya Suwabe, Junichi Hoshino, Noriko Hayami
- The University of Tokyo (Department of Nephrology and Endocrinology), Masaomi Nangaku, Tetsuhiro Tanaka, Yoshifumi Hamasaki, Kenjiro Honda

- The University of Tokyo(Department of Pediatrics), Yutaka Harita, Shoichiro Kanda, Yuko Kajiho
- University of Tsukuba (Department of Nephrology), Kunihiro Yamagata, Joichi Usui, Tetsuya Kawamura
- Yokohama City University Graduate School of Medicine (Department of Medical Science and Cardiorenal Medicine), Kouichi Tamura, Hiromichi Wakui, Tomohiko Kanaoka, Ryu Kobayashi
- Yokohama City University Medical Center, Nobuhito Hirawa, Sanae Saka, Akira Fujiwara

Koushinetsu District

- Niigata University Graduate School of Medical and Dental Sciences (Division of Clinical Nephrology and Rheumatology), Ichiei Narita, Shin Goto, Yumi Itoh, Naofumi Imai
- Shinshu University School of Medicine (Department of Nephrology), Yuji Kamijo, Koji Hashimoto, Akinori Yamaguchi, Harada Makoto
- University of Yamanashi Hospital (Third Department of Internal Medicine), Kazuya Takahashi, Fumihiko Furuya

Hokuriku District

- National Hospital Organization Kanazawa Medical Center (Department of Nephrology and Rheumatology), Kiyoki Kitagawa
- Kanazawa Medical University School of Medicine (Department of Nephrology), Hitoshi Yokoyama, Kengo Furuichi, Keiji Fujimoto, Norifumi Hayashi
- Kanazawa Medical University (Department of Diabetology & Endocrinology), Daisuke Koya, Munehiro Kitada, Yuka Kuroshima
- Kanazawa University Hospital (Division of Nephrology), Takashi Wada, Miho Shimizu, Norihiko Sakai, Yasunori Iwata
- Komatsu Sophia Hospital, Yasuhiro Katou, Yuta Yamamura
- Koshino Internal Medicine Clinic, Yoshitaka Koshino
- Public Central Hospital of Matto-Ishikawa, Chikako Takaeda
- Sugita Genpaku Memorial Obama Municipal Hospital, Haruyoshi Yoshida, Takayasu Horiguchi
- Toyama Prefectural Central Hospital (Department of Internal Medicine), Yasuyuki Shinozaki, Masahiko Kawabata
- Toyama City Hospital (Department of Internal Medicine), Satoshi Ota, Yoh-ichi Ishida
- University of Fukui, Faculty of Medical Sciences (Department of Nephrology), Masayuki Iwano, Naoki Takahashi, Kenji Kasuno, Daisuke Mikami
- University of Toyama (Second Department of Internal Medicine), Hidenori Yamazaki

Tokai District

- Aichi Children's Health and Medical Center (Department of Pediatric Nephrology), Naoya Fujita, Satoshi Hibino, Kazuki Tanaka

・Aichi Medical University School of Medicine (Division of Nephrology and Rheumatology), Yasuhiko Ito, Takuhito Nagai, Takayuki Katsuno, Hironobu Nobata

・Chuno Kosei Hospital, Shogo Kimura, Yuka Soga

・Fujinomiya City General Hospital, Masanori Sakakima

・Fujita Health University School of Medicine (Department of Nephrology), Yukio Yuzawa, Naotake Tsuboi, Hiroki Hayashi, Kazuo Takahashi

・Hamamatsu University School of Medicine, University Hospital (Internal Medicine1, Division of Nephrology), Hideo Yasuda, Naro Ohashi, Taichi Sato

・Japanese Red Cross Nagoya Daini Hospital (Kidney Center), Asami Takeda, Yasuhiro Otsuka

・Nagoya City East Medical Center, Minamo Ono, Tatsuya Tomonari

・Nagoya City University Graduate School of Medical Sciences (Department of Cardio-Renal Medicine and Hypertension), Michio Fukuda, Masashi Mizuno, Taisei Suzuki, Satoru Kominato

・Nagoya Kyoritsu Hospital (Department of Internal Medicine), Hirotake Kasuga

・Nagoya University Graduate School of Medicine (Department of Nephrology), Shoichi Maruyama, Yoshinari Yasuda, Tomoki Kosugi, Takuji Ishimoto

・Shizuoka General Hospital (Department of Nephrology), Noriko Mori, Satoshi Tanaka

・Mie University Graduate School of Medicine (Department of Cardiology and Nephrology), Tomohiro Murata, Mika Fujimoto, Kan Katayama

・Japan Community Health care Organization Yokkaichi Hazu Medical Center (Division of Nephrology and Blood Purification), Yasuhide Mizutani, Hitoshi Kodera, Masato Miyake

Kinki District

・Hyogo Prefectural Nisihinomiya Hospital (Department of Pathology), Kazumasa Oka

・Hyogo Prefectural Kobe Children's Hospital (Department of Nephrology), Hiroshi Kaito

・Ikeda City Hospital (Department of Nephrology), Nobuyuki Kajiwara

・Kitano Hospital, Tazuke Kofukai Medical Research Institute (Department of Nephrology and Dialysis), Tatsuo Tsukamoto, Tomomi Endo, Eri Muso

・Kobe University Graduate School of Medicine (Division of Nephrology and Kidney Center), Shinichi Nishi, Shunsuke Goto

・Kobe University Graduate School of Medicine (Department of Pediatrics), Kazumoto Iijima, Kandai Nozu, Tomoko Horinouchi

・Japan Community Health care Organization Kobe Central Hospital, Yoko Adachi, Takaaki Nishihara, Michitsugu Kamezaki

・National Hospital Organization Kyoto Medical Center (Division of Nephrology), Koichi Seta

・Kyoto Prefectural University of Medicine Graduate School of Medical Science (Department of Nephrology), Keiichi Tamagaki, Tetsuro Kusaba, Yayoi Shiotsu

・Kyoto University Graduate School of Medicine (Department of Nephrology), Motoko Yanagita, Hideki Yokoi, Kaoru Sakai, Akira Ishii

・Nara Medical University (Department of Nephrology), Kazuhiko Tsuruya, Kenichi Samejima

・National Cerebral and Cardiovascular Center (Division of Hypertension and Nephrology), Fumiki Yoshihara

・National Hospital Organization Osaka National Hospital (Department of Nephrology), Hirotsugu Iwatani

・Osaka City University Graduate School of Medicine (Department of Nephrology), Katsuhito Mori, Akihiro Tsuda, Shinya Nakatani

・Osaka City General Hospital (Division of Nephrology and Hypertension), Yoshio Konishi, Takashi Morikawa, Chizuko Kitabayashi

・Osaka City General Hospital (Division of Pediatrics), Rika Fujimaru

・Osaka General Medical Center (Department of Kidney Disease and Hypertension), Terumasa Hayashi, Tatsuya Shoji

・Osaka Women's and Children's Hospital (Department of Pediatric Nephrology and Metabolism), Katsusuke Yamamoto

・Osaka Medical College (Department of Pediatrics), Akira Ashida

・Osaka Red Cross Hospital (Department of Nephrology), Akira Sugawara, Masao Koshikawa, Yoshihisa Ogawa, Tomoko Kawanishi

・Osaka Rosai Hospital (Department of Nephrology), Atsushi Yamauchi, Katsuyuki Nagatoya, Daisuke Mori, Ryota Haga

・Osaka University Graduate School of Medicine (Department of Nephrology), Yoshitaka Isaka, Ryohei Yamamoto, Tomoko Namba-Hamano

・Saiseikai Shiga Hospital (Division of Nephrology), Toshiki Nishio

・Shiga University of Medical Science (Department of Medicine), Shinichi Araki

・Shirasagi Hospital (Kidney Center), Shigeichi Shoji, Kenjiro Yamakawa, Senji Okuno

・Toyonaka Municipal Hospital (Division of Nephrology), Megumu Fukunaga

・Wakayama Medical University (Department of Pediatrics), Yuko Shima,

・Wakayama Medical University (Department of Nephrology), Takashi Shigematsu, Masaki Ohya

Chugoku District

・Kawasaki Medical School (Department of Nephrology and Hypertension), Naoki Kashihara, Tamaki Sasaki, Hajime Nagasu

・Kurashiki Central Hospital (Division of Nephrology), Kenichiro Asano, Motoko Kanzaki, Kosuke Fukuoka

・Hiroshima University Hospital (Department of Nephrology), Takao Masaki, Shigehiro Doi, Ayumu Nakashima, Toshiki Doi

・Mizushima Kyodo Hospital (Department of Nephrology), Kan Yamazaki, Nobuyoshi Sugiyama, Yuichiro Inaba, Kouji Ozeki

・Okayama Saiseikai General Hospital (Department of Nephrology), Makoto Hiramatsu, Keisuke

Maruyama, Noriya Momoki

- Okayama University Graduate School of Medicine, Dentistry and Pharmaceutical Sciences (Department of Nephrology, Rheumatology, Endocrinology and Metabolism), Jun Wada, Hiroshi Morinaga, Ayu Akiyama, Yasuhiro Onishi
- Okayama University Graduate School of Medicine, Dentistry and Pharmaceutical Sciences (Department of Pediatrics), Hiroyuki Miyahara
- Saiseikai Yamaguchi General Hospital (Department of Internal Medicine), Tsuyoshi Imai
- Shimane University Faculty of Medicine (Division of Nephrology), Takafumi Ito, Masahiro Egawa, Shohei Fukunaga
- Tottori University, Faculty of Medicine (Division of Pediatrics and Perinatology), Shinichi Okada, Koichi Kitamoto, Hiroki Yokoyama, Yuko Yamada

Shikoku District

- Kagawa University, Faculty of Medicine (Department of Cardiorenal and Cerebrovascular Medicine & Department of Clinical Pathology), Tadashi Sofue, Tetsuo Minamino, Emi Ibuki
- Kochi University, Kochi Medical School (Department of Endocrinology, Metabolism and Nephrology), Yoshio Terada, Taro Horino, Yoshiko Shimamura,Tatsuki Matsumoto
- Kochi University, Kochi Medical School (Department of Pediatrics), Mikiya Fujieda, Masayuki Ishihara, Yoshiki Nagao
- Tokushima University Graduate School (Department of Pediatrics, Institute of Biomedical Sciences), Shoji Kagami, Maki Urushihara, Yukiko Kinoshita
- Tokushima University Graduate School (Department of Nephrology, Institute of Biomedical Sciences), Hideharu Abe, Kojiro Nagai

Kyushu District

- Fukuoka University (Division of Nephrology and Rheumatology, Department of Internal Medicine, Faculty of Medicine), Kosuke Masutani, Tetsuhiko Yasuno, Kenji Ito
- Japanese Red Cross Fukuoka Hospital (Department of Pediatrics), Ken Hatae, Manao Nishimura, Hiroyo Maruyama, Miyu Aoyama
- Japanese Red Cross Fukuoka Hospital (Nephrology and Dialysis Center), Koji Mitsuiki
- Kumamoto University Graduate School of Medical Sciences (Department of Nephrology), Masashi Mukoyama, Masataka Adachi
- Kurume University School of Medicine (Division of Nephrology, Department of Medicine), Kei Fukami, Junko Yano
- Kyushu University Graduate School of Medical Sciences (Department of Medicine and Clinical Science), Toshiaki Nakano, Akihiro Tsuchimoto, Yuta Matsukuma, Kenji Ueki
- Kyushu University Graduate School of Medical Sciences (Department of Environmental Medicine), Yutaka Kiyohara, Toshiharu Ninomiya, Masaharu Nagata
- Miyazaki Prefectural Miyazaki Hospital (Division of Nephrology), Naoko Yokota-Ikeda, Keiko

Kodama

- Nagasaki University Hospital (Department of Pathology), *late* Takashi Taguchi
- Nagasaki University Hospital (Department of Nephrology), Tomoya Nishino, Kenta Torigoe, Kiyokazu Tsuji
- National Hospital Organization Fukuokahigashi Medical Center (Division of Nephrology), Yusuke Kuroki
- National Hospital Organization Kyushu Medical Center, Masaru Nakayama
- Oitaken Kouseiren Tsurumi Hospital (Division of Nephrology), Ryokichi Yasumori
- Saga University, Faculty of Medicine (Department of Internal Medicine), Motoaki Miyazono, Tsuyoshi Takashima, Shuichi Rikitake, Makoto Fukuda
- St. Mary's Hospital, Harumichi Higashi
- University of Miyazaki Hospital (Division of Nephrology), Shouichi Fujimoto, Masao Kikuchi, Shoko Ochiai
- University of Occupational and Environmental Health (Second Department of Internal Medicine), Masahito Tamura, Tetsu Miyamoto
- University of the Ryukyus Graduate School of Medicine (Department of Cardiology, Nephrology and Neurology), Yusuke Ohya, Kentaro Kohagura

Supplemental Table S1. Rare comorbidities of mitochondrial nephropathy

	Type of gene mutations					total (n=81)
	mtDNA point mutation (n=66)	mtDNA single delition (n=3)	mtDNA multiple deletion (n=3)	nDNA mutation related to CoQ10		
				COQ2/COQ6 (n=3)	*COQ8b* (n=6)	
**Comorbidities during all observation period, n (%)**						
Retinitis pigmentosa	1 (1.5)	2 (66.7)	0 (0)	0 (0)	0 (0)	3 (3.7)
Optic atrophy	2 (3.0)	0 (0)	0 (0)	0 (0)	0 (0)	2 (2.5)
Pancreatitis	1 (1.5)	1 (33.3)	0 (0)	0 (0)	0 (0)	2 (2.5)
Arrythmia	5 (7.6)	2 (66.7)	0 (0)	0 (0)	1 (16.7)	8 (9.9)
Liver dysfunction	3 (4.6)	2 (66.7)	2 (66.7)	0 (0)	0 (0)	7 (8.6)
Growth hormone deficiency	3 (4.6)	0 (0)	0 (0)	0 (0)	0 (0)	3 (3.7)
Hypothyroidism	2 (3.0)	0 (0)	1 (33.3)	2 (66.7)	0 (0)	5 (6.2)
Hypoparathyroidism	0 (0)	2 (66.7)	1 (33.3)	0 (0)	0 (0)	3 (3.7)
Depression	2 (3.0)	0 (0)	0 (0)	0 (0)	0 (0)	2 (2.5)
Autism	2 (3.0)	0 (0)	0 (0)	0 (0)	0 (0)	2 (2.5)

mtDNA, mitochondrial DNA; nDNA, nuclear DNA; CoQ10, coenzyme Q10

Supplemental Table S2. Clinicopathological features of mitochondrial nephropathy by differences in genetic analysis

Characteristics	All cases (n=113)	Genetic analysis		
		Cases with gene mutation confirmed as causative mutation of mitochondrial disease by a genetic analysis (n=81)	Cases without gene mutation confirmed as causative mutation of mitochondrial disease by a genetic analysis (n=20)	Cases that did not receive a genetic analysis (n=12)
**The onset of renal manifestations, n (%)***				
Childhood (0-19 years)	60 (53.1)	42 (51.9)	12 (60.0)	6 (50.0)
Adulthood (≥20 years)	53 (46.9)	39 (48.1)	8 (40.0)	6 (50.0)
Elevated lactate level, n (%)**	44 (48.9)	28 (43.8)	11 (61.1)	5 (62.5)
**Familly history, n (%)**				
Mitochondrial disease	44 (48.9)	39 (48.1)	5 (25.0)	0 (0)
Diabetes	49 (43.4)	43 (53.1)	4 (20.0)	2 (16.7)
Cases with a kidney biopsy, n (%)	80 (70.8)	57 (70.4)	13 (65.0)	10 (83.3)
**Data at kidney biopsy, median (IQR)**				
eGFR (mL/min/1.73 m^2)	78.8 (44.6-99.4)	80.9 (46.9-107.5)	57.1 (31.1-82.2)	83.7 (52.2-96.8)
Proteinuria (g/gCre)	1.51 (0.60-3.30)	1.81 (0.90-4.20)	1.24 (0.44-2.28)	1.23 (0.41-3.18)
**Kidney biopsy diagnosis, n (%)**				
Focal segmental glomerulosclerosis	39 (48.8)	30 (52.6)	4 (30.8)	5 (50.0)
Nephrosclerosis	9 (11.3)	6 (10.5)	2 (15.4)	1 (10.0)
Diabetic nephropathy	8 (10.0)	7 (12.3)	1 (7.7)	0 (0)
Tubulointerstitial nephropathy	6 (7.5)	3 (5.3)	3 (23.1)	0 (0)
Minor glomerular abnormality	11 (13.8)	6 (10.5)	1 (7.7)	4 (40.0)
Unknown	7 (8.8)	5 (8.8)	2 (15.4)	0 (0)
Abnormal findings of mitochondrial under EM, n (%)***	46 (57.5)	34 (59.7)	7 (53.9)	5 (50.0)
**Cell type with abonormal findings of mitochondria, n (%)**				
Podocytes	32 (40.0)	25 (43.9)	3 (23.1)	4 (40.0)
Tubular cells	27 (33.8)	21 (36.8)	4 (30.8)	2 (20.0)
Other (endothelial, mesangial, smooth muscle)	7 (8.8)	4 (7.0)	1 (7.7)	2 (20.0)
Granular swollen epithelial cells, n (%)	18 (22.5)	13 (22.8)	3 (23.1)	2 (20.0)

IQR, interquartile range; mtDNA, mitochondrial DNA; nDNA, nuclear DNA; CoQ10, coenzyme Q10

*Time at the first detection of at least one of following 3 renal manifestations, proteinuria (≥0.15 g/gCre), reduced eGFR (<60 mL/min/1.73 m^2) or Fanconi syndrome, is defined as the onset timing of renal manifestations.

**The denominator for the percentage calculation is the number of cases in which measurements of lactate levels were performed. Elevated lactate level was defined as serum or cerebrospinal fluid lactate levels over 2 mmol/L (18 mg/dL) or detection of lactate peak on brain magnetic resonance spectroscopy.

***Abnormal findings of mitochondrial are defined as an increase in the number or abnormal morphology of mitochondria.
